# Screening of shared molecular markers between cervical cancer and major depressive disorder and the mechanism of CRAT/CLIC4 regulating EMT in cervical cancer cells

**DOI:** 10.1186/s12885-026-16571-5

**Published:** 2026-07-20

**Authors:** Hua-Hua Zhang, Min Liu, Ya-Ni Chen, Ming-Ru Zhang, Jing Zhang, Jing He

**Affiliations:** 1 Medical Research and Experimental Center, Yan’an Medical College, Yan’an University, Yan’an, Shaanxi Province China; 2 Department of Pathology, Yan’an University Affiliated Hospital, Yan’an, Shaanxi Province China; 3 Medical Research and Experimental Center, The Second Affiliated Hospital of Xi’an Medical University, Xi’an, Shaanxi Province China; 4 Department of Gynecology, Yan’an University Affiliated Hospital, No. 19 Fenghuang Street, Baota District, Shaanxi Province Yan’an City, China

**Keywords:** Cervical cancer, Major depressive disorder, Hub gene, Epithelial-mesenchymal transition

## Abstract

**Background:**

Cervical cancer (CC) ranks among the most prevalent malignant neoplasms affecting women worldwide. Tumor recurrence, distant metastases, and chemotherapy resistance significantly hinder long-term clinical survival and therapeutic outcomes. Clinical studies indicate a heightened prevalence of depressive symptoms and major depressive disorder (MDD) among CC patients, suggesting the possibility of bidirectional adverse biological interactions between cervical tumor progression and depressive states. However, the shared molecular signatures underlying both cervical carcinoma and depressive disorders have yet to be fully elucidated, highlighting the need for identifying reliable molecular markers for supplementary diagnosis and prognostic stratification of CC.

**Methods:**

Two datasets (GSE98793 for MDD and GSE63514 for CC) were retrieved from the Gene Expression Omnibus (GEO) database to identify shared differentially expressed genes (DEGs). Gene clustering analysis of the overlapping DEGs was conducted utilizing Metascape. Venn analysis facilitated the identification of overlaps between shared DEGs and extracellular vesicle (EV)-related genes. Six machine learning algorithms were employed to pinpoint core comorbid genes and develop a diagnostic model. Functional enrichment, drug sensitivity, and survival analyses were performed to assess gene functions, therapeutic potential, and prognostic relevance. In vitro experiments provided additional validation of the effects of core genes on CC malignant phenotypes and epithelial-mesenchymal transition (EMT). Transcriptomic and clinical data of cervical cancer were retrieved from TCGA. Corrected Pearson correlation analysis and PROGENy pathway activity analysis were performed via R packages to dissect the pathway regulatory correlations of *CRAT* and *CLIC4*.

**Results:**

A total of 85 intersecting DEGs were identified from the transcriptomes of CC and female MDD. Gene cluster analysis indicated that these genes are primarily linked to biological processes such as immune homeostasis modulation, cell cycle progression, PD-1/PD-L1 immune checkpoint signaling, and dopaminergic synaptic transmission. Machine learning techniques identified four candidate hub genes-*EZR*, *MAOA*, *CLIC4*, and *CRAT*. This four-gene signature exhibited a high discriminatory capacity for CC and moderate diagnostic performance for MDD samples. Bioinformatic predictions implied that these genes are involved in tumor metabolic reprogramming regulation, cell cycle control, EMT activation, and anti-tumor immune responses. Cobimetinib was recognized as a potential small-molecule agent targeting *CLIC4*, *EZR*, and *MAOA*. Survival analysis indicated that *CRAT* may function as a favorable prognostic marker, whereas increased expression of *CLIC4* correlates with adverse clinical outcomes. Risk scores derived from the four hub genes could serve as independent prognostic indicators for CC. In vitro cellular experiments partially confirmed that *CRAT* knockdown enhances malignant transformation and EMT in CC cells, while *CLIC4* interference inhibits cell proliferation, migration, and EMT progression. Multi-pathway correlation networks demonstrated a positive association between *CRAT* and the JAK-STAT signaling cascade. Linear correlation analysis further substantiated that *CRAT* expression was significantly positively correlated with the levels of *JAK1* and *STAT3*. Concurrently, *CLIC4* showed a positive correlation with the TGF-β signaling pathway, and single-gene correlation analysis confirmed a pronounced linear positive association between *CLIC4* and its downstream molecules, *TGFBR1* and *SMAD2*.

**Conclusion:**

This study examines the shared molecular signatures between CC and MDD. The four-gene model, comprising *EZR*, *MAOA*, *CLIC4*, and *CRAT*, shows promise for differential diagnosis and independent prognostic assessment of cervical cancer. In vitro experiments indicate that elevated *CRAT* expression is associated with extended survival, whereas increased *CLIC4* expression is linked to a poor prognosis. These two genes influence malignant phenotypes and EMT through distinct pathways. This research provides preliminary evidence of molecular crosstalk between the two disorders and offers a theoretical foundation for the identification of prognostic biomarkers and therapeutic targets for CC.

**Supplementary Information:**

The online version contains supplementary material available at 10.1186/s12885-026-16571-5.

## Introduction

Cervical cancer (CC) remains to represent a significant global public health challenge, ranking as the fourth most commonly diagnosed malignancy and the fourth leading cause of cancer-related mortality among females worldwide [1]. Despite the extensive application of combined therapeutic strategies, including surgery, chemoradiotherapy, and targeted therapy, challenges such as tumor recurrence, distant metastasis, and chemotherapy resistance continue to impede long-term patient survival, with only minimal improvements observed in the 5-year overall survival (OS) rate [2]. Thus, there is an urgent necessity to investigate the molecular regulatory mechanisms underlying CC and to identify clinically viable diagnostic biomarkers and therapeutic targets.

Recent clinical data indicate a substantial correlation between psychological status, particularly depressive symptoms, and the onset, progression, and clinical prognosis of tumors. Numerous omics studies have revealed overlapping molecular alterations in both CC and depressive disorders. These alterations encompass abnormalities in various genetic loci, signaling pathways, and biological processes, including inflammatory imbalances, oxidative stress overload, dysregulation of apoptosis, disrupted neurotransmitter metabolism, and aberrant energy metabolism [3]. By investigating the intersecting genes altered in both CC and depressive disorder samples, a deeper understanding of the molecular changes common to both pathological conditions can be achieved. More importantly, this cross-disease transcriptomic analysis presents a novel approach for uncovering potential diagnostic and prognostic biomarkers for CC.

With advancements in high-throughput sequencing and bioinformatics, the integrated analysis of multiple transcriptomic datasets has emerged as a key method for identifying disease-associated molecular signatures and elucidating molecular regulatory mechanisms [4]. The utilization of machine learning algorithms enhances the reliability and precision of filtering hub candidate genes [5]. However, a notable gap exists in research that integrates multi-transcriptomic data with machine learning to systematically identify intersecting signature genes affected in CC and depressive disorders. Additionally, the biological functions of these candidate molecules in the malignant progression of CC remain insufficiently understood, and relevant cellular experimental evidence is limited, necessitating further comprehensive investigation.

This study integrated two independent transcriptomic datasets to identify intersecting differentially expressed genes (DEGs) associated with CC and depressive disorders. Various machine learning algorithms were employed to filter for key hub genes. Comprehensive computational analyses, including survival assessments, immune microenvironment evaluations, and drug susceptibility predictions, were conducted to assess the diagnostic accuracy, prognostic significance, and targeted therapeutic potential of these candidate genes. Furthermore, loss- and gain-of-function assays performed in CC cell lines validated the biological roles and downstream mechanisms of *CLIC4* and *CRAT*, focusing on their regulatory effects on cell proliferation, migration, and epithelial-mesenchymal transition (EMT). This research aims to clarify shared molecular signatures between CC and depressive disorders, providing an experimental foundation for developing prognostic biomarkers and personalized targeted therapies for CC.

## Methods

### Data collection

Datasets relevant to gene expression were retrieved from the Gene Expression Omnibus (GEO) database. The dataset designated as GSE98793, associated with major depressive disorder (MDD), comprised 192 peripheral blood samples. For the purpose of conducting a sex-stratified analysis, individuals with comorbid anxiety were excluded, resulting in a subset of 96 samples from females (48 healthy controls and 48 MDD patients) and 32 samples from males (16 healthy controls and 16 MDD patients). Additionally, the dataset labeled GSE63514, related to CC, contained 128 cervical tissue specimens. From this dataset, 24 normal cervical samples and 28 invasive CC samples were selected, excluding those exhibiting cervical intraepithelial neoplasia.

### Identification of differentially expression genes (DEGs) and functional enrichment analysis

DEGs from MDD and CC expression profiles were identified utilizing the limma package in R. Tissue-specific threshold criteria were employed, reflecting the distinct transcriptional characteristics inherent to each dataset. For the peripheral blood-derived MDD dataset (GSE98793), where peripheral blood demonstrates mild transcriptional fluctuations under chronic inflammation, a lenient cutoff (|log2FC| > 0.5, *P* < 0.05) was implemented to capture subtle yet consistent neuroimmune transcriptional changes and to retain low-abundance, disease-related transcripts. Conversely, for the cervical tumor tissue-based CC dataset (GSE63514), where malignant transformation induces significant transcriptional reprogramming with marked gene expression alterations, a stringent threshold (|log2FC| > 1, adj.P.Val < 0.05) was applied to filter out background noise and ensure the identification of reliable tumor-specific DEGs. Uniform standardized criteria (adjusted *P* ≤ 0.05, |log2FC| ≥ 0.2 for MDD; adjusted *P* ≤ 0.05, |log2FC| ≥ 1 for CC) were subsequently applied for further analyses. DEGs were visualized using volcano plots generated with the ggplot2 package. A three-way Venn diagram was plotted to screen genes specifically shared by female MDD (MDD_F) and CC, excluding overlapping DEGs from male MDD (MDD_M). Functional clustering of intersecting DEGs was performed using Metascape (v3.5.20240901, https://metascape.org) [6]. Ultimately, these candidate genes were cross-referenced with differentially expressed proteins isolated from extracellular vesicles (EVs) in the brain tissues of MDD patients to identify potential key comorbidity genes [7].

### Integrated machine learning screening, model construction and performance analysis

The machine learning analysis was systematically divided into three distinct and sequential stages, each with well-defined boundaries. In Stage 1, LASSO regression was applied to overlapping DEGs to identify key features and reduce the dimensionality of the candidate gene set. Stage 2 involved a random and stratified division of the dataset into a 70% training set and a 30% internal test set. Six machine learning algorithms—LASSO, random forest (RF), decision tree (DT), XGBoost, gradient boosting machine (GBM), and support vector machine (SVM)—were employed to construct models based on the training set. In Stage 3, the independent internal test set was utilized for evaluating the models’ performance using the area under the receiver operating characteristic curve (ROC-AUC), ensuring that no test set data was used during feature selection or model training. Importantly, the study did not include external clinical cohorts for cross-cohort validation. An integrated approach employing all six algorithms was specifically used to identify stable core variables through intersection selection [8]. Lasso regression was subsequently utilized to ascertain the optimal number of features, leveraging cross-validation based on binomial deviance. Feature importance was assessed using RF, XGBoost, and GBM methods. A DT model was then developed using the core variables, with optimization achieved through cross-validation of the complexity parameter and tree size, while the classification rules were visualized. Model performance was evaluated using metrics such as cross-validated root mean square error (RMSE) and out-of-bag error.

### Diagnostic evaluation of candidate biomarkers

To assess the differential diagnostic potential of the candidate genes *MAOA*, *EZR*, *CRAT*, and *CLIC4* in distinguishing CC from MDD, receiver operating characteristic (ROC) curves were constructed based on gene expression levels. The corresponding area under the curve (AUC) values, along with 95% confidence intervals (95% CI), were calculated concurrently. Stratified random sampling was employed to partition the dataset for internal validation, dividing the samples into a training set and an internal test set in a 7:3 ratio. All statistical analyses were conducted using the pROC package in the R programming language. The discriminative performance of each individual gene, as well as the combined four-gene model, was evaluated separately within the CC and MDD cohorts.

### Functional enrichment of key genes and analysis of the tumor immune microenvironment

Gene set enrichment analysis (GSEA) was conducted using the Hallmark gene set collection to identify biological pathways associated with the four significant genes (*MAOA*, *EZR*, *CRAT*, *CLIC4*) in CC. The tumor immune microenvironment was profiled by employing multiple immune infiltration algorithms, including TIMER, CIBERSORT, EPIC, and MCP counter, to evaluate correlations between the expression of these four genes and levels of immune cell infiltration. Additionally, correlation analyses assessed the associations between the four genes and the expression of chemokines, chemokine receptors, immune-activating genes, immunosuppressive genes, and major histocompatibility complex (MHC) genes in tumor tissues.

### Drug sensitivity and molecular intersection analysis of key genes in CC

The Genomics of Drug Sensitivity in Cancer (GDSC) database was utilized to investigate how the expression levels of the four key genes (*MAOA*, *EZR*, *CRAT*, *CLIC4*) relate to sensitivity to anticancer drugs. Drugs with a *P*-value of less than 0.001 were identified as having a significant correlation. A four-set Venn diagram was employed to analyze the distribution and intersections of molecular sets linked to the genes *CLIC4*, *CRAT*, *EZR*, and *MAOA*. A statistical significance threshold of *P* < 0.01 was applied to evaluate the reliability of the intersection results and elucidate the overlapping relationships among the sets and core shared molecules.

### Prognostic value of key genes and *cox* regression analysis in CC

The study also explored the relationship between the expression levels of the four specific genes (*MAOA*, *EZR*, *CRAT*, *CLIC4*) and OS in CC patients using the Kaplan-Meier plotter database. To assess the independent prognostic significance of clinical variables—including age, clinical stage, histological grade, and TNM stage—alongside a risk score derived from the expression levels of these four genes, both univariate and multivariate Cox regression models were utilized.

### Single-gene pathway activity and associated gene analysis of CRAT and CLIC4 in CC

STAR-counts RNA-seq and clinical data for CC samples were obtained from the TCGA GDC portal (https://portal.gdc.cancer.gov). The TPM matrices underwent log₂(TPM + 1) normalization, and 306 samples with comprehensive sequencing and clinical profiles were retained for analysis. Pearson correlation analysis was performed to assess the transcriptome-wide expression associations between *CRAT* and *CLIC4*, with multiple testing corrections applied using the Benjamini-Hochberg method. The R package “easier” was employed to calculate PROGENy activity scores for 14 tumor-related pathways, which were subsequently analyzed for correlations between target genes and pathway activities. Correlation scatter plots were generated using the “ggstatsplot” package for visualization purposes [9, 10].

### Cell experiment

#### Cell culture

Human CC cell lines HeLa and CaSki, along with the human normal cervical epithelial cell line H8, were obtained from the Medical Research Experiment Center of the Medical College. Both H8 and HeLa cells were maintained in RPMI-1640 supplemented with 10% fetal bovine serum (FBS) at 37 ℃ under 5% CO_2_. CaSki cells were maintained in DMEM supplemented with 10% FBS at 37 ℃ under 5% CO_2_.

### Small-interfering RNA, plasmid synthesis, and transfection

Two specific small interfering RNAs targeting *CLIC4* (siCLIC4-1, siCLIC4-2) and a negative control siRNA (siNC) were designed and synthesized by GenePharma Co., Ltd. (Shanghai, China). The sequences are as follows: siCLIC4-1 sense: 5’-GGAGUUGUAUUUAGUGUGATT-3’, antisense: 5’-UCACACUAAAUACAACUCCTT-3’; siCLIC4-2 sense: 5’-GGU GGU GGC CAA AAA AUA UTT-3’, antisense: 5’-AUAUUUUUUGGCCACCACCTT-3’; siNC sense: 5’-UUC UCC GAA CGU GUCACGUTT-3’, antisense: 5’-ACGUGACACGUUCGGAGAATT-3’. The *CRAT* overexpression vector (over-CRAT) and its corresponding empty vector control were constructed by Wuhan Miaoling Biotechnology Co., Ltd. CC cells were transfected with siRNAs using the Polyplus transfection system and with plasmids using the Lipo8000 transfection system, respectively.

### Western blotting

Total cellular protein was extracted 48 h post-transfection using RIPA lysis buffer, followed by concentration determination using the BCA method. SDS-PAGE was employed to separate equal amounts of protein samples, which were subsequently transferred to PVDF membranes. The membranes were blocked with 5% skim milk at room temperature for one hour and then incubated overnight at 4 ℃ with the appropriate primary antibodies. Following washing, the membranes were incubated with horseradish peroxidase-conjugated secondary antibodies for one hour at room temperature, followed by visualization with ECL chemiluminescence. Immunoreactive bands were visualized using the Real-time quantitative Western Blot detection and analysis system LuQAS1000 (Real-Blot, Xi’an, China). The gray values of protein bands were quantified using ImageJ software, with β-actin serving as the internal reference for calculating relative expression levels. The primary antibodies utilized included: CRAT (Proteintech, 15170-1-AP, dilution ratio of 1:1000), CLIC4 (ProMab, P17913, dilution ratio of 1:1000), E-cadherin (Proteintech, 20874-1-AP, dilution ratio of 1:1000), N-cadherin (Proteintech, 22018-1-AP, dilution ratio of 1:1000), Vimentin (Proteintech, 10336-1-AP, dilution ratio of 1:1000), Snail1 (Proteintech, 26183-1-AP, dilution ratio of 1:1000), and β-actin (Proteintech, 66009-1-Ig, dilution ratio of 1:10000).

### Isolation of RNA, creation of cDNA, and quantitative real-time PCR (qRT-PCR)

RNA extraction, cDNA synthesis, and qPCR analysis were conducted as previously described [11].The primers were synthesized by Sangon Biotech (Shanghai) Co., Ltd., with the following sequences specified as follows: *CLIC4* Forward: 5’-TGA AAG CAT AGG AAA CTG CCC-3’, Reverse: 5’-GGT CAA CAG TCG TCA CAC TAA A-3’; *CRAT* Forward: 5’-ATT CTG ACA GGT GAA GCC TC-3’, Reverse: 5’-CTT CAG GTA GTG GTC CAG G-3’; *GAPDH* Forward: 5’-TGA AGG TCG GAG TCA ACG GAT T-3’, Reverse: 5’-CCT GGA AGA TGG TGA TGG GAT T-3’. The relative mRNA expression levels of *CLIC4* and *CRAT* were determined using the 2^−ΔΔCt^ method, with normalization to the internal reference gene *GAPDH*.

### CCK-8 assay

The HeLa and CaSki cells were independently seeded into 96-well culture plates. At intervals of 24, 48, and 72 h post-transfection, 10 µL of CCK-8 solution was added to each well, followed by a 2-hour incubation period at 37 ℃. Subsequently, the absorbance at 450 nm was then quantified using a molecular devices multi-mode microplate reader.

### Colony formation assay

Twenty-four hours after transfection, a density of 800 cells per well was utilized to seed HeLa and CaSki cells into 6-well plates, which were then cultured at 37 ℃ with 5% CO_2_ for 7–10 days until visible cell clones were observed. Following two washes with PBS, cells were fixed using 4% paraformaldehyde for 30 min and stained with 0.1% crystal violet for an additional 30 min. After rinsing and air drying, colony images were captured.

### Transwell migration assay

Twenty-four hours post-transfection, HeLa and CaSki cells were introduced into the upper chambers with medium containing medium with 1% FBS, while 600 µL of medium with 10% FBS was placed in the lower chambers. Cells were incubated for 48 h at 37 ℃ in a 5% CO₂ environment, then fixed with 4% paraformaldehyde for 20 min and stained with 0.1% crystal violet for 30 min. Wet imaging was employed to capture images.

### Statistical analysis

Statistical analyses were performed using SPSS version 26.0 and GraphPad Prism version 10.0 software. Data following a normal distribution were presented as mean ± standard deviation (mean ± SD). The independent-sample t-test was utilized for two-group comparisons, while one-way ANOVA with post hoc tests was employed for multiple group comparisons. A *P*-value of less than 0.05 was considered statistically significant.

## Results

### Identification and functional enrichment analysis of DEGs in CC and female MDD

**Fig. 1:**
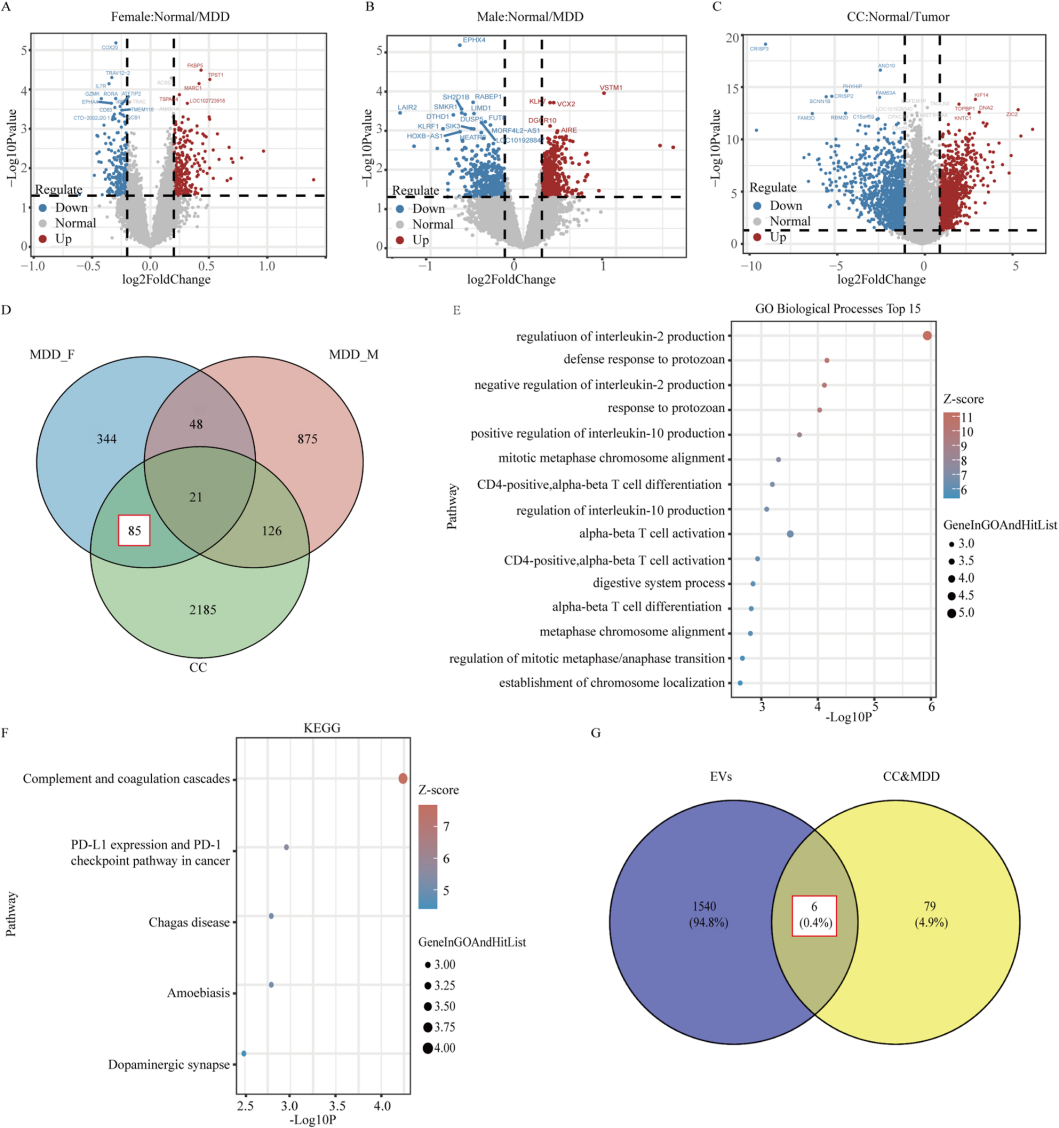
Screening of differentially expressed genes and related functional enrichment analysis in CC and MDD. **A-C** Visual representations of differentially expressed genes(DEGs) using volcano plots in **(A)** female MDD patients versus normal controls, (**B**) male MDD patients versus normal controls, and (**C**) cervical cancer (CC) tissues versus normal cervical tissues. **D** Venn diagram depicting the shared DEGs among female MDD and CC patient (excluding DEGs related to male MDD), with 85 target DEGs finally obtained. **E** GO enrichment analysis results of 85 target DEGs. **F** KEGG enrichment analysis results of 85 target DEGs. **G** A Venn diagram presenting the shared EV-related genes and the 85 DEGs common to both CC and MDD (CC&MDD), to explore potential EV-related mechanisms among the shared genes

Upon excluding patients with comorbid anxiety from the female MDD cohort in dataset GSE98793, differential expression analysis revealed a significant downregulation of *COX20*, *IL7R*, and *EPHX4*, alongside notable upregulation of *FKBP5*, *MARC1*, and *TSPT1* (Fig. 1A). In the male MDD cohort, after excluding individuals with comorbidities, a marked downregulation of immune-related genes such as *SH2D1B*, *RABEP1*, *EPHX4*, and *LAIR2* was observed. Concurrently, functional genes including *KLK7*, *DGCR10*, *VCX2*, and *VSTM1* showed upregulation (Fig. 1B). In the CC dataset GSE63514, a differential expression analysis was performed on 24 normal cervical tissue samples and 28 CC tissue samples. This analysis indicated a significant upregulation of genes involved in mitosis and cell cycle homeostasis regulation, such as *KIF14*, *TOPBP1*, *DNA2*, and *ZIC2*, in CC tissues. Conversely, genes exhibiting tissue-specific expression patterns and known functional roles, including *ANO10*, *FAM63A*, *PHYHIP*, and *SCNN1B*, were significantly downregulated (Fig. 1C). A Venn diagram was constructed to identify the intersection of DEGs between female patients with MDD and those with CC, while excluding DEGs from male MDD patients. This analysis identified 85 target DEGs for further investigation (Fig. 1D). Gene Ontology (GO) analysis indicated that these DEGs were primarily involved in immune regulation (e.g., interleukin regulation, T cell activation) and cell cycle progression (e.g., mitotic chromosome alignment) (Fig. 1E). Kyoto Encyclopedia of Genes and Genomes (KEGG) enrichment analysis demonstrated significant enrichment of these genes in pathways such as the complement and coagulation cascades, the PD-1/PD-L1 immune checkpoint pathway, and the dopaminergic synapse pathway (Fig. 1F). Furthermore, the intersection of DEGs with genes associated with EVs was analyzed using another Venn diagram, which showed minimal overlap between the two gene sets: 1,540 genes (94.8%) were exclusive to the EV gene set, 79 genes (4.9%) were unique to the intersecting gene set of CC and depressive disorder, and only 6 genes (0.4%) were common to both groups (Fig. 1G).

### Machine learning-based identification and validation of key feature genes

**Fig. 2:**
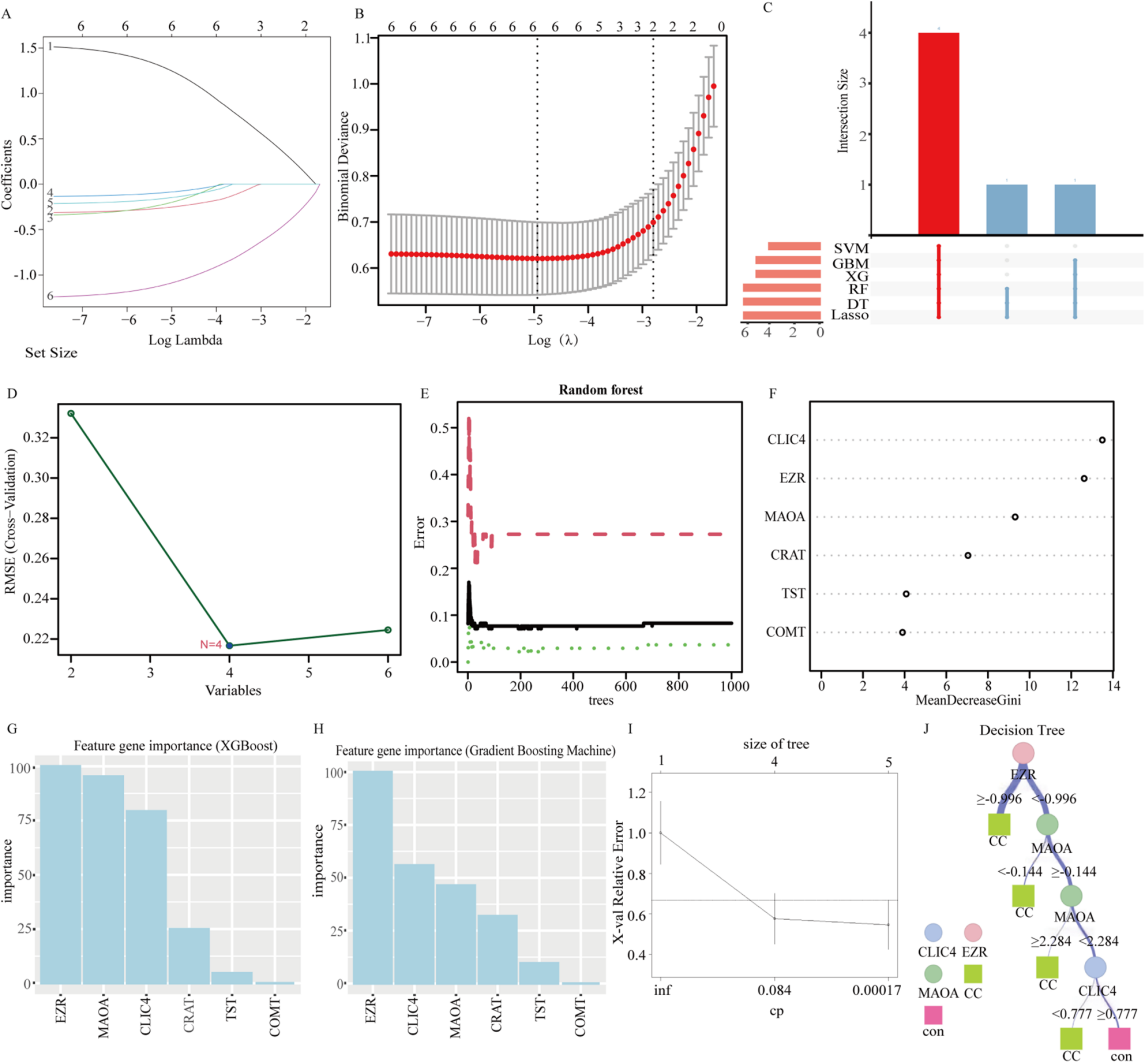
Identification of key feature genes based on machine learning. **A** Lasso regression coefficient paths for six candidate genes across different log(λ) values. **B** Binomial deviance curve for Lasso regression, where the vertical dashed line indicates the optimal λ value. **C** Intersection analysis of feature genes selected by six machine learning models (Lasso, SVM, Random Forest, XGBoost, Gradient Boosting Machine, and Decision Tree). **D** Root mean square error (RMSE) derived from 10‑fold cross‑validation, demonstrating the optimal number of feature variables (*N* = 4). **E** Random Forest model error curve plotted against the number of decision trees. **F–H** Feature importance rankings of the six candidate genes from (**F**) Random Forest (measured by mean decrease in Gini), (**G**) XGBoost, and (**H**) Gradient Boosting Machine. **I** Cross-validation error curve for decision tree optimization, with the optimal complexity parameter (cp. = 0.084) highlighted. **J** Visualization of the final decision tree constructed using key genes (EZR, MAOA) as splitting nodes to classify control and case groups

To identify stable molecular markers for CC, various machine learning algorithms were employed for feature screening and validation based on the transcriptomic profiles of cervical lesions. Initially, lasso regression was used for dimensionality reduction of differentially expressed proteins, resulting in the identification of six candidate genes: *EZR*, *MAOA*, *COMT*, *CRAT*, *TST*, and *CLIC4* (Fig. 2A and B). Subsequently, an integration of six algorithms—lasso, RF, DT, XGBoost, GBM, and SVM—was used for feature intersection analysis, which culminated in the identification of four common core genes: *EZR*, *MAOA*, *CRAT*, and *CLIC4* across different models (Fig. 2C). Cross-validation utilizing SVM indicated that the 4-gene signature (*EZR*, *MAOA*, *CRAT*, *CLIC4*) provided the optimal balance between model performance and generalization ability (Fig. 2D), thereby corroborating the intersection results. Further evaluation of feature importance was conducted using RF, XGBoost, GBM, and DT models. The mean decrease Gini ranking from RF indicated that *CLIC4*, *EZR*, *MAOA*, and *CRAT* were the top four ranked genes (Fig. 2E and F). Both the XGBoost and GBM models demonstrated that *EZR*, *MAOA*, *CLIC4*, and *CRAT* contributed significantly, whereas *TST* and *COMT* showed minimal contributions (Fig. 2G and H). Following the pruning of the DT model, the optimal tree size was established at four, with the feature importance ranking further emphasizing the central role of these four genes (Fig. 2I and J). In summary, multi-algorithm screening, intersection validation, and feature importance evaluation consistently identified *EZR*, *MAOA*, *CRAT*, and *CLIC4* as reliable core molecular signatures of CC.

### Diagnostic potential value of candidate biomarkers in patients with CC and MDD

**Fig. 3:**
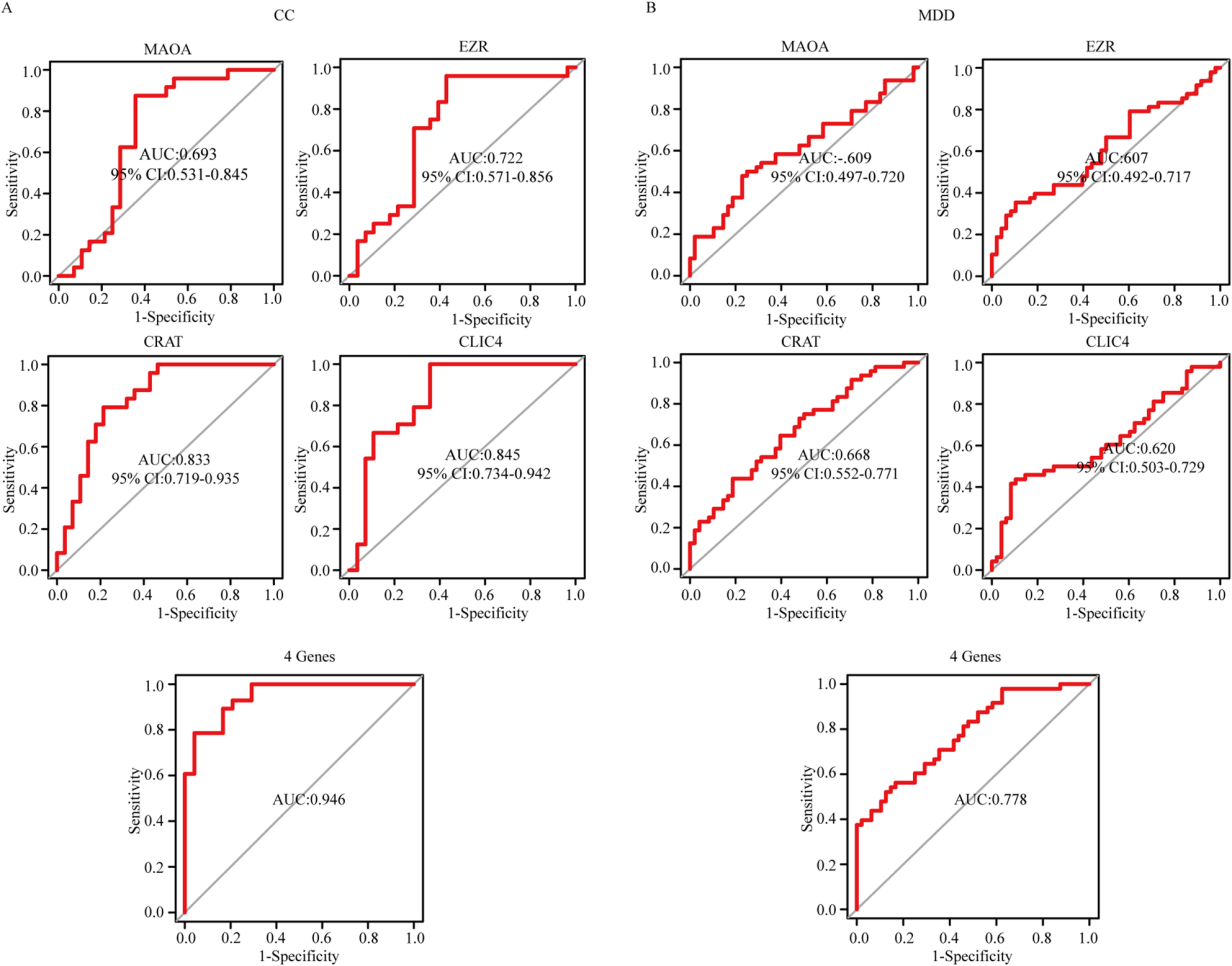
Diagnostic potential of MAOA, EZR, CRAT, and CLIC4 in patients with CC and MDD. **A** ROC curves for individual genes (MAOA, EZR, CRAT, CLIC4) and the four-gene combined model in the CC cohort. **B** ROC curves for individual genes (MAOA, EZR, CRAT, CLIC4) and the four-gene combined model in the MDD cohort

In the CC samples, the AUC values for the individual candidate genes *CLIC4*, *CRAT*, *EZR*, and *MAOA* were 0.845 (95% CI: 0.734–0.942), 0.833 (95% CI: 0.719–0.935), 0.722 (95% CI: 0.571–0.856), and 0.693 (95% CI: 0.531–0.845), respectively. Among these genes, *CLIC4* and *CRAT* demonstrated favorable diagnostic efficacy. The combined model incorporating all four genes achieved an AUC of 0.946, indicating excellent discriminatory capacity for CC (Fig. 3A). When applied to transcriptome samples of MDD, the diagnostic performance of each gene remained moderate, with *CRAT* exhibiting an AUC of 0.668 (95% CI: 0.552–0.771), *CLIC4* an AUC of 0.620 (95% CI: 0.503–0.729), *MAOA* an AUC of 0.609 (95% CI: 0.497–0.720), and *EZR* an AUC of 0.607 (95% CI: 0.492–0.717). Importantly, the integration of these four genes into a combined panel yielded an AUC of 0.778, significantly surpassing the performance of any individual gene and demonstrating moderate discriminatory capacity for identifying depressive disorder samples (Fig. 3B).

### Functional enrichment and immune microenvironment correlation of the four key genes in CC

**Fig. 4:**
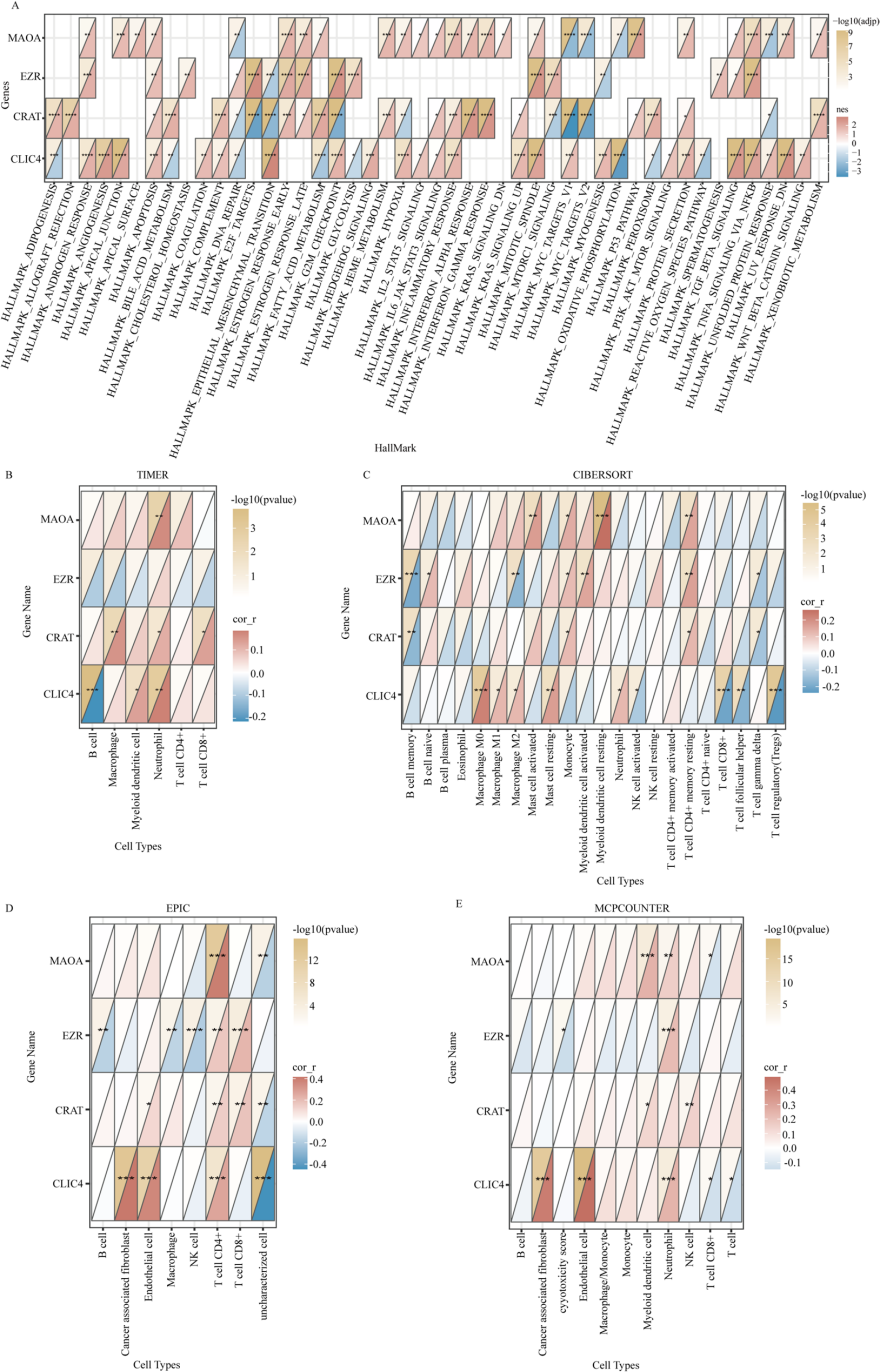
Functional enrichment and immune infiltration correlation analysis of MAOA, EZR, CRAT, and CLIC4 in CC. **A** Hallmark gene set enrichment analysis (GSEA) heatmap illustrating the correlation between the expression of four target genes and hallmark signaling pathways. **B-E** Correlation analysis between the four genes and immune cell infiltration in CC, evaluated using four distinct algorithms: (**B**) TIMER, (**C**) CIBERSORT, (**D**) EPIC, and (**E**) MCP counter. *P < 0.05, **P < 0.01, ***P < 0.001, t-test. r: Pearson correlation coefficient

GSEA indicated that the gene signature comprising *CLIC4*, *CRAT*, *EZR*, and *MAOA* was significantly enriched across multiple signaling pathways intricately associated with tumor progression. Within metabolic and stress response pathways, this quartet of genes was involved in adipogenesis and fatty acid metabolism, with *CLIC4* specifically enriched in pathways related to angiogenesis. Additionally, concerning cell cycle regulation and EMT, the gene signature exhibited associations with EMT signaling and mitotic spindle assembly. Furthermore, all four genes were enriched in pathways related to apoptosis and DNA repair, suggesting their collective involvement in the regulation of various tumor-related biological processes (Fig. 4A). To investigate the immunoregulatory roles of *EZR*, *MAOA*, *CRAT*, and *CLIC4* within the tumor microenvironment of CC, four computational algorithms—TIMER, CIBERSORT, EPIC, and MCP-counter—were employed to assess the correlation between gene expression and the abundance of infiltrating immune cells. The findings indicated that *CLIC4* demonstrated significant correlations with various immune cell populations, whereas *EZR* exhibited moderate correlations. In contrast, *MAOA* and *CRAT* displayed only weak associations with infiltrating immune cells (Fig. 4B). The analysis of immune characteristics suggested that *EZR* was associated with chemokines and molecules involved in immune activation and suppression, while *CLIC4* was positively correlated with immune-suppressive genes such as PD-L1, highlighting its pivotal role in antigen presentation and the immunosuppressive microenvironment (Supplementary Fig. 1A-1D).

### Correlation between four key genes and anticancer drug sensitivity in CC

**Fig. 5:**
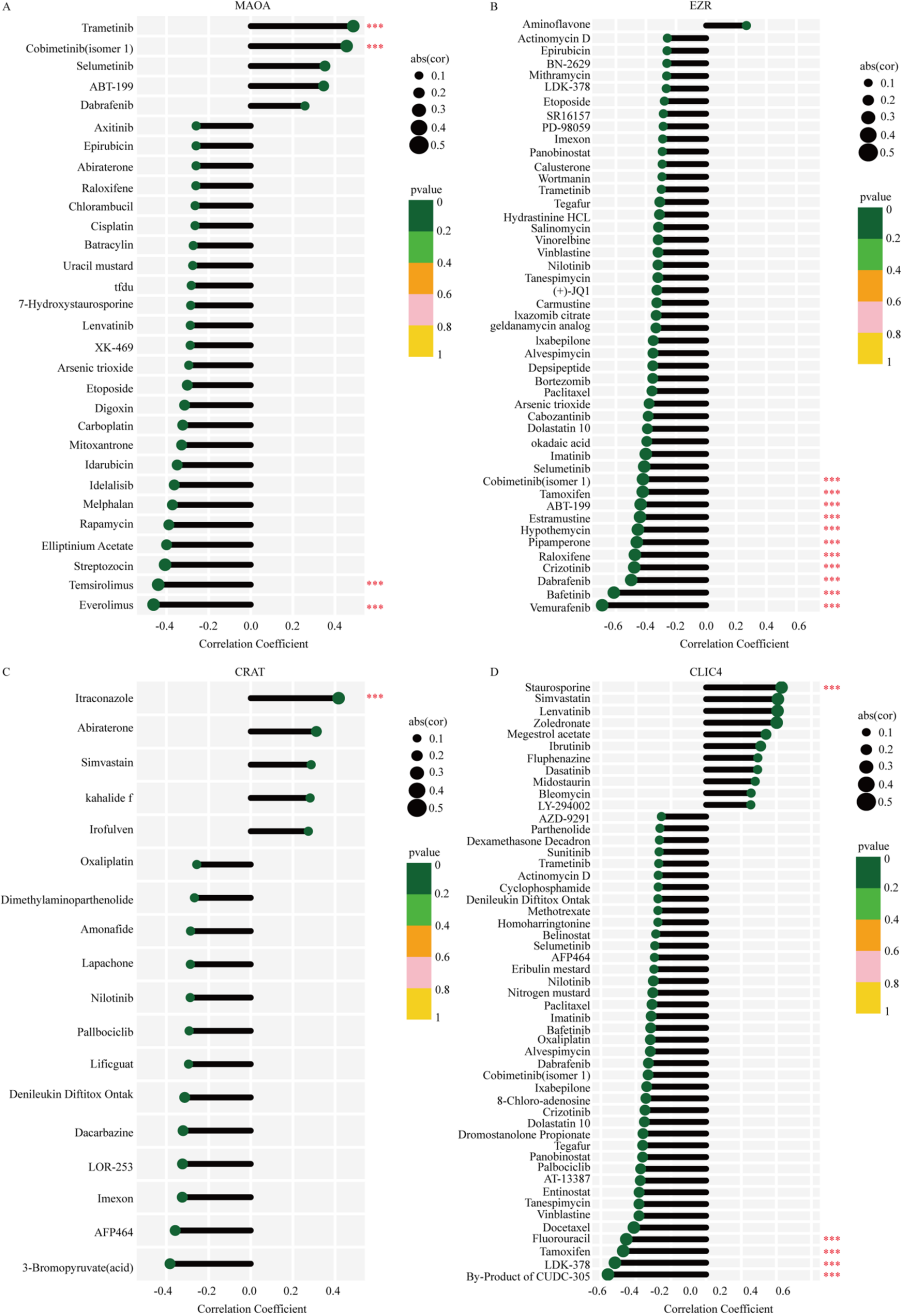
Analysis of drug sensitivity correlation for MAOA, EZR, CRAT, and CLIC4 in CC cell lines. **A-D** Forest plots showing the correlation between the expression of (**A**) MAOA, (**B**) EZR, (**C**) CRAT, and (**D**) CLIC4 with the sensitivity of various anti-cancer drugs. ***P < 0.001, t-test

The results of the drug sensitivity analysis indicated that the expression of *MAOA* exhibited a positive correlation with MEK pathway-targeted drugs, trametinib and cobimetinib, while showing a negative correlation with mTOR inhibitors, temsirolimus and everolimus (Fig. 5A). Conversely, the expression level of *EZR* displayed a negative correlation with sensitivity to various antitumor drugs, including MAPK/RAF-MEK pathway inhibitors (such as cobimetinib, vemurafenib, dabrafenib, and hypothemycin), tyrosine kinase inhibitors (including crizotinib and bafetinib), as well as selective estrogen receptor modulators (such as tamoxifen and raloxifene) (Fig. 5B). *CRAT* was positively correlated with itraconazole, suggesting its role in predicting responses to antifungal and metabolic therapies (Fig. 5C). Furthermore, *CLIC4* expression was positively correlated with staurosporine sensitivity, and negatively correlated with tamoxifen, fluorouracil, and other agents (Fig. 5D). This four-gene signature has the potential to aid in predicting drug responses and guiding personalized therapy in CC. Venn diagram analysis revealed that the numbers of unique elements within the four sets—*CLIC4*, *EZR*, *MAOA*, and *CRAT*—were 16 (33.3%), 14 (29.2%), 8 (16.7%), and 3 (6.3%), respectively, indicating a generally low degree of overlap among the sets. A single unique common element, cobimetinib (isomer 1), was identified among the *CLIC4*, *EZR*, and *MAOA* sets. This finding suggests that cobimetinib may serve as a potential candidate for targeting the pathways associated with *CLIC4*, *EZR*, and *MAOA* concurrently (Supplementary Fig. 2).

### Survival analysis of four key genes and their prognostic value in CC

**Fig. 6:**
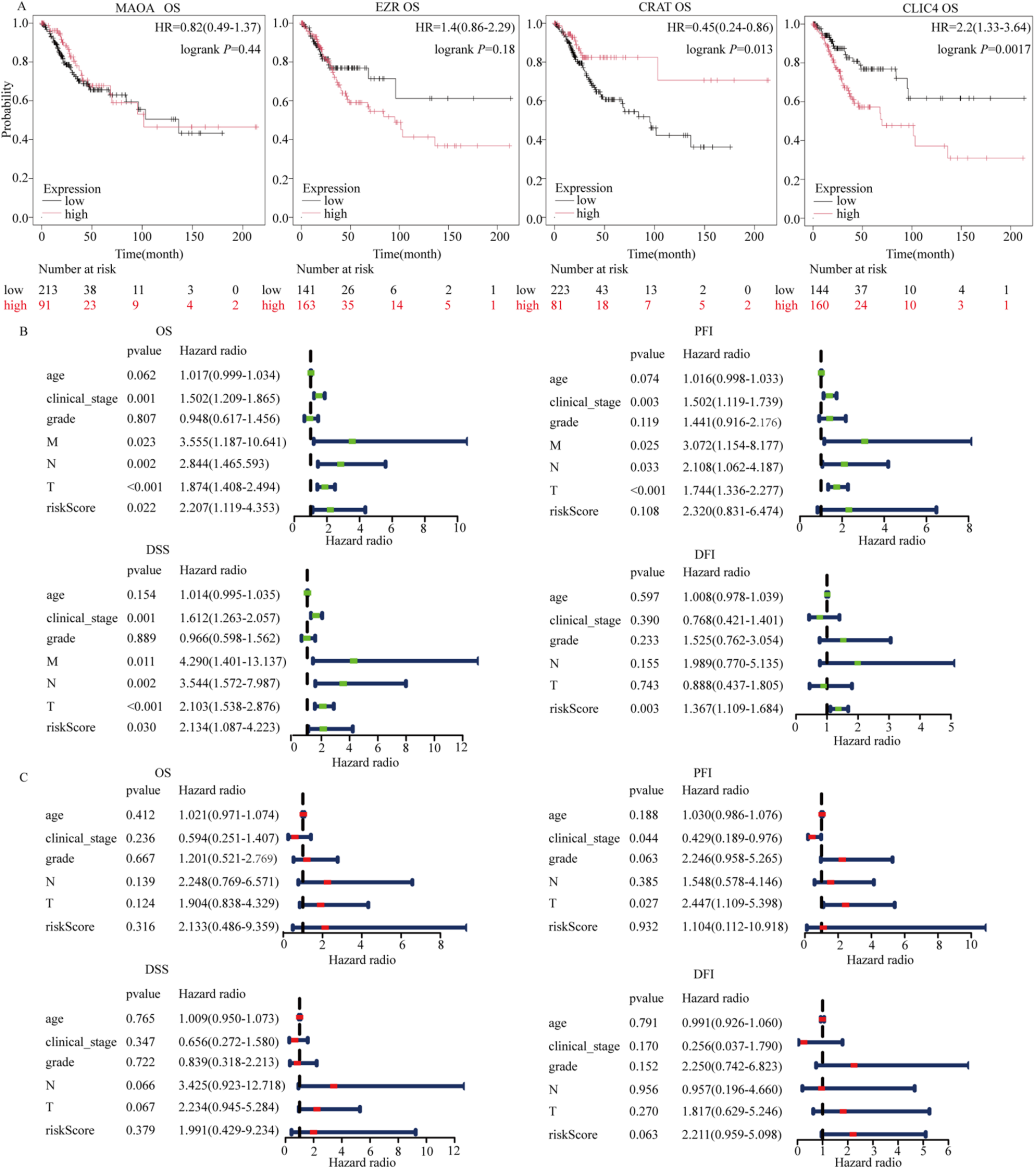
Survival analysis of four key genes and their prognostic value in CC. **A** Kaplan-Meier overall survival (OS) curves stratified by high versus low expression of MAOA, EZR, CRAT, and CLIC4. **B** Forest plots from multivariate *Cox* regression analyses evaluating the prognostic impact of clinical variables and the four-gene risk score on OS, progression free interval (PFI), disease specific survival (DSS), and disease free interval (DFI). **C** Univariate Cox regression forest plots showing the prognostic effects of clinical features and riskScore on OS, PFI, DSS, and DFI, with hazard ratios (HR) and 95% confidence intervals (CI) annotated

Survival analysis utilizing the Kaplan-Meier plotter database demonstrated that the four genes investigated exhibited markedly distinct prognostic characteristics. Specifically, elevated expression of *CRAT* was identified as a protective factor, while increased expression of *CLIC4* significantly correlated with reduced OS. In contrast, the expression levels of *MAOA* and *EZR* did not show a significant association with patients’ OS (Fig. 6A). After adjusting for clinical confounding variables, multivariate *Cox* regression analysis revealed that advanced T, N, and M stages were linked to decreased OS, disease-specific survival (DSS), and progression-free interval (PFI). The risk score emerged as an independent adverse prognostic factor for both OS and DSS, and was the sole independent factor influencing the disease-free interval (DFI), although it did not demonstrate a significant association with PFI (Fig. 6B). Importantly, none of the variables achieved statistical significance in the univariate analysis (Fig. 6C), indicating that the prognostic effects of the genes in question were likely obscured by clinical confounding factors.

### Effects of CRAT overexpression on proliferation, migration and EMT of CC cells

**Fig. 7:**
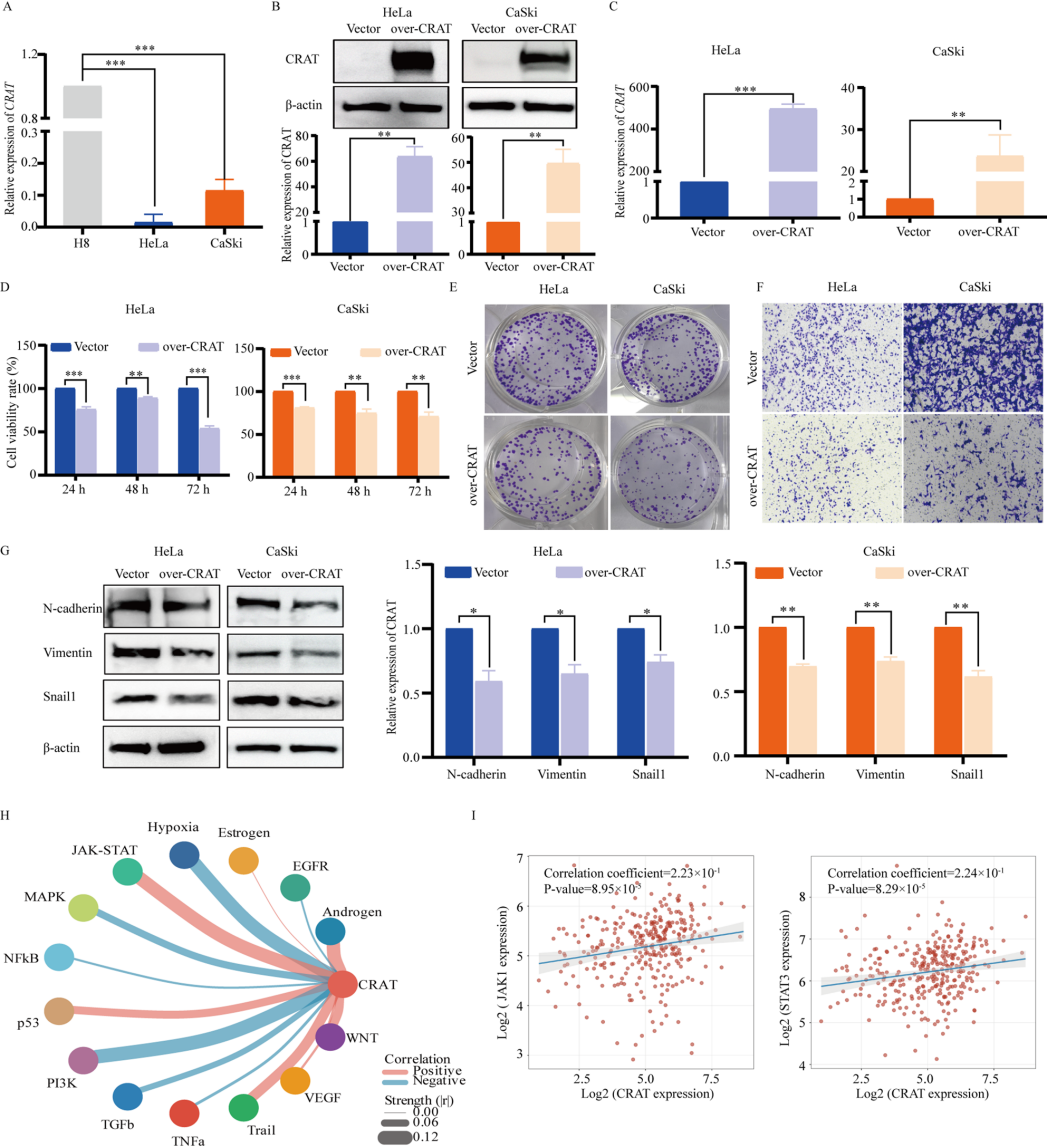
Overexpression of CRAT inhibits proliferation, migration, and EMT process of CC cells. **A** qRT-PCR was used to detect the expression level of *CRAT* in normal cervical epithelial cells H8 and CC cells HeLa and CaSki. **B-C** Western blotting and qRT-PCR verified the overexpression efficiency of CRAT in HeLa and CaSki cells. **D** CCK-8 assay was used to detect the effect of *CRAT* overexpression on the viability of HeLa and CaSki cells. **E** Colony formation assay was used to detect the effect of *CRAT* overexpression on the colony formation ability of CC cells. **F** Transwell migration assay was used to detect the effect of *CRAT* overexpression on the migration ability of CC cells (crystal violet staining, ×100). **G** Western blotting was used to detect the effect of CRAT overexpression on the expression of EMT-related proteins (N-cadherin, Vimentin, Snail1) in CC cells. **H** Network diagram showing correlations between *CRAT* and canonical tumor signaling pathways. **I** Scatter plots illustrating the correlations between *CRAT* and *JAK1*/*STAT3* expression in CC samples. Data represented mean ± SD. *P < 0.05, **P < 0.01, ***P < 0.001 (one-way ANOVA, Student’s t-test)

qRT-PCR analysis indicated a significant reduction in the relative expression of *CRAT* in HeLa and CaSki cells compared to the normal cervical epithelial cell line H8 (Fig. 7A). To elucidate the role of *CRAT* in CC, a *CRAT* overexpression plasmid was constructed and transfected into HeLa and CaSki cells. Western blotting and qRT-PCR analyses confirmed that the protein and mRNA expression levels of CRAT were significantly elevated in the overexpression group (over-CRAT) compared to the empty vector control group (Vector) (Fig. 7B and C), thereby validating the successful establishment of the overexpression model. The CCK-8 assay demonstrated that *CRAT* overexpression led to a significant reduction in cell viability at 24, 48, and 72 h in both HeLa and CaSki cells relative to the Vector group (Fig. 7D). These findings suggest a marked inhibition of the proliferative capacity of CC cells due to *CRAT* overexpression. Results from the colony formation assay revealed that *CRAT* overexpression significantly diminished the colony-forming capacity of HeLa and CaSki cells compared to the Vector group (Fig. 7E). Similarly, the transwell migration assay indicated a significant reduction in the number of migrated cells in the *CRAT*-overexpressing group relative to the Vector group (Fig. 7F), indicating that *CRAT* overexpression compromises both colony formation and migratory abilities of CC cells. Western blotting analysis further demonstrated that CRAT overexpression led to a significant downregulation of EMT-related markers, including N-cadherin, Vimentin, and Snail1, in HeLa and CaSki cells compared to the Vector group (Fig. 7G). Functional enrichment analysis revealed a significant enrichment of *CRAT* within the JAK/STAT3 signaling pathway. The multi-pathway correlation network (Fig. 7H) illustrated a positive correlation between *CRAT* and the JAK-STAT pathway. To elucidate this association further, a single-gene correlation analysis was conducted using transcriptomic data from CC samples within the TCGA cohort (Fig. 7I). The findings indicated a significant and linear positive correlation between *CRAT* expression and the expression levels of *JAK1* and *STAT3*, suggesting that *CRAT* is closely associated with the core molecular activation of the JAK/STAT3 signaling pathway in CC.

### CLIC4 knockdown regulates biological behaviors and expression of EMT-related markers in CC cells

**Fig. 8:**
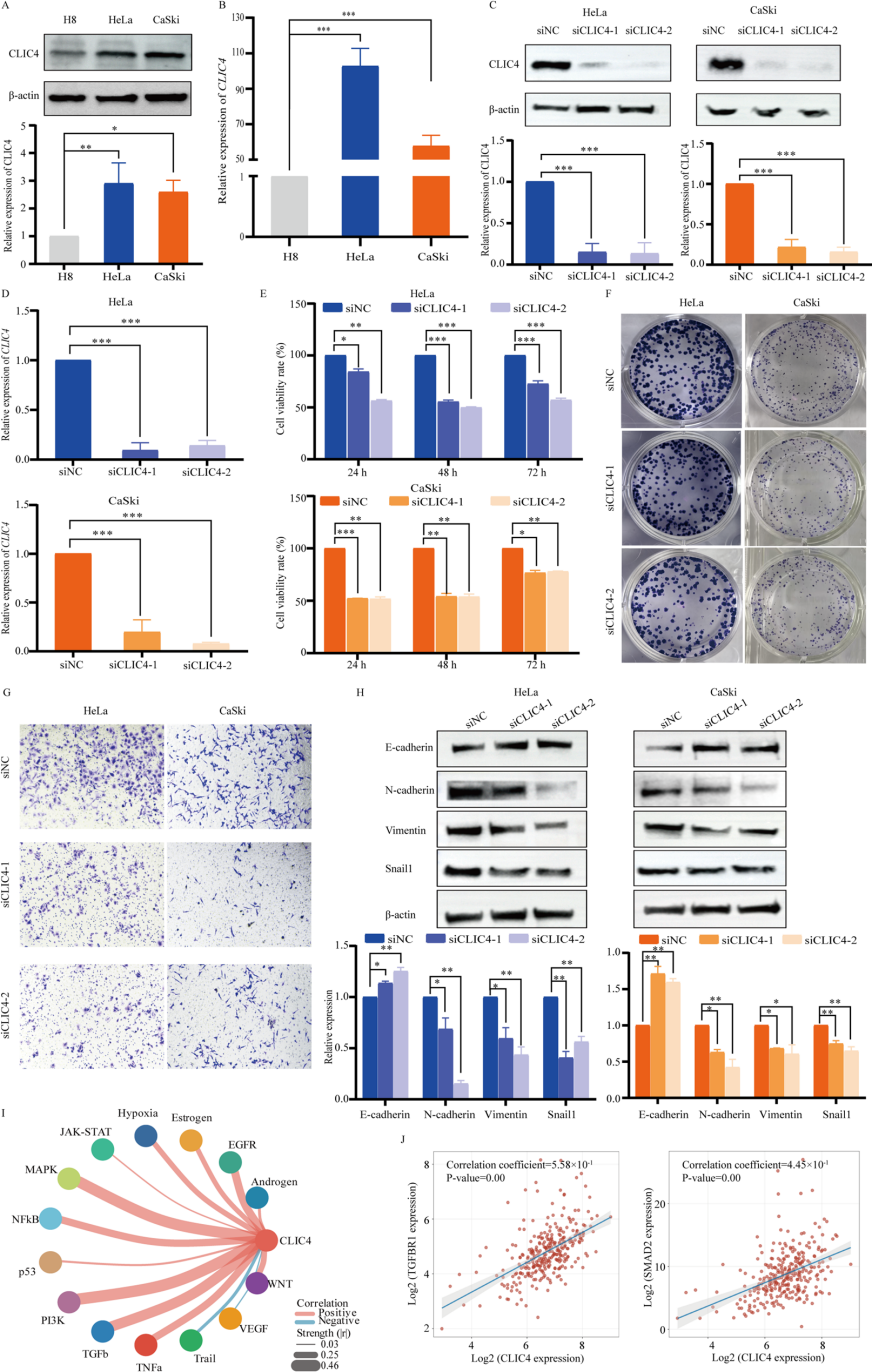
Knockdown of *CLIC4* inhibits proliferation, migration and reverses EMT process in CC cells. **A** Western blotting was used to detect the protein expression of CLIC4 in normal cervical epithelial cells H8 and CC cells Hela, Caski, and quantitative analysis of gray value of relative expression of CLIC4 protein. **B** qRT-PCR was used to detect the mRNA expression of *CLIC4* in normal cervical epithelial cells H8 and CC cells Hela, Caski. **C** Western blotting was used to detect the knockdown efficiency of CLIC4 in HeLa and CaSki cells after siRNA transfection, and quantitative analysis of gray value of CLIC4 knockdown efficiency. **D** qRT-PCR was used to detect the knockdown efficiency of CLIC4 in HeLa and CaSki cells after siRNA transfection. **E** CCK-8 assay was used to detect the effect of *CLIC4* knockdown on the proliferative viability of HeLa and CaSki cells. **F** Colony formation assay was used to detect the effect of *CLIC4* knockdown on the colony formation ability of HeLa and CaSki cells. **G** Transwell migration assay was used to detect the effect of *CLIC4* knockdown on the migration ability of HeLa and CaSki cells (crystal violet staining,×200). **H** Western blotting was used to detect the expression changes and quantitative analysis of EMT-related markers (E-cadherin, N-cadherin, Vimentin, Snail1) after CLIC4 knockdown. **I** Network diagram showing correlations between *CLIC4* and canonical tumor signaling pathways. **J** Scatter plots illustrating the correlations between *CLIC4* and *TGFBR1*/*SMAD2* expression in CC samples. Data represented mean ± SD. *P < 0.05, **P < 0.01, ***P < 0.001 (one-way ANOVA)

The expression level of CLIC4 in H8 and CC cells (HeLa and CaSki) was initially assessed. Western blotting analysis revealed a significant elevation in CLIC4 protein expression in HeLa and CaSki cells compared to H8 cells (Fig. 8A and B), suggesting a potential oncogenic role for CLIC4 in the pathogenesis of CC. To investigate the function of *CLIC4*, *CLIC4*-specific siRNAs (siCLIC4-1, siCLIC4-2) were designed and transfected into HeLa and CaSki cells, with siNC serving as the control. Forty-eight hours post-transfection, the siCLIC4 groups exhibited a significant decrease in CLIC4 protein and mRNA expression levels, as assessed by Western blotting and qRT-PCR analyses, compared to the siNC group (Fig. 8C and D). CCK-8 assay results indicated that the proliferative viability of HeLa and CaSki cells was significantly reduced following *CLIC4* knockdown relative to the siNC group (Fig. 8E). Additionally, colony formation assays demonstrated that *CLIC4* knockdown markedly decreased colony formation in HeLa and CaSki cells (Fig. 8F). The Transwell migration assay further revealed that the number of transmembrane cells in the siCLIC4 groups was significantly reduced compared to the siNC group (Fig. 8G), highlighting that *CLIC4* knockdown significantly attenuates the migration potential of CC cells. To understand the molecular processes through which *CLIC4* affects CC cell migration, expression variations of markers associated with EMT were analyzed. Western blotting analysis indicated that CLIC4 knockdown resulted in a marked increase in E-cadherin, accompanied by a significant decrease in N-cadherin, Vimentin, and Snail1 (Fig. 8H). Functional enrichment analysis revealed a significant enrichment of *CLIC4* within the TGF-β signaling pathway. The multi-pathway correlation network (Fig. 8I) illustrated a positive correlation between *CLIC4* and the TGF-β pathway. To elucidate this association further, a single-gene correlation analysis indicated a significant and linear positive correlation between *CLIC4* expression and the expression levels of *TGFBR1* and *SMAD2* (Fig. 8J), suggesting that *CLIC4* is closely associated with the core molecular activation of the TGF-β signaling pathway in CC.

## Discussion

Existing molecular and epidemiological research has established that depression may facilitate the onset and malignant progression of CC by inducing systemic immune dysfunction, sustaining chronic inflammation, and disrupting the regulatory pathways associated with persistent HPV infection [12–14]. Conversely, CC and its clinical treatments, including radiotherapy and chemotherapy, can lead to physical pain, reproductive impairment, and various adverse reactions, thereby precipitating depressive symptoms in patients. These negative emotional states further diminish treatment adherence and exacerbate physical discomfort, creating a mutually detrimental interaction between CC and depression. A cross-sectional study of CC patients reported a 13.5% prevalence of comorbid depression within this population, providing clinical evidence for the correlation between these two disorders [15]. Despite the identification of clinical correlations and overlapping regulatory pathways between CC and depression, the fundamental molecular signatures connecting these two conditions remain inadequately understood. This study adopts a cross-disease transcriptomic approach to identify four key hub genes (*MAOA*, *EZR*, *CRAT*, and *CLIC4*) exhibiting dysregulated expression in both CC and MDD. In vitro functional assays further confirmed that *CLIC4* is overexpressed in CC and promotes oncogenic activity, while *CRAT* is underexpressed and functions as a tumor suppressor. Both genes influence the malignant phenotypes of CC cells, including proliferation and migration, through the regulation of the EMT signaling pathway.

Initially, a differential expression analysis was conducted, identifying 85 intersecting genes exhibiting synchronous transcriptional dysregulation in both female MDD and CC. This observation aligns with findings from Mendelian randomization studies, which have established a causal relationship between these two conditions and have reported numerous overlapping dysregulated molecules [3]. Building on this gene set, cross-tissue molecular screening was performed in conjunction with multiple machine learning algorithms, ultimately identifying *EZR*, *MAOA*, *CRAT*, and *CLIC4* as core associated biomarkers. Despite the inherent heterogeneity between peripheral blood and cervical tumor tissues, these tissues exhibit intrinsic biological interconnections. Peripheral monocytes reflect systemic immune and neuroendocrine disruptions induced by depression, while cervical tissues are indicative of local epithelial malignant transformation and remodeling of the tumor microenvironment. Peripheral circulation serves as a conduit for transmitting central stress signals to solid tumors, thereby establishing a biological foundation for homologous molecular perturbations and pathological interactions between these two tissue types. Both tissues are involved in a highly interconnected regulatory cascade: chronic depression persistently activates the hypothalamic-pituitary-adrenal (HPA) axis, initiates chronic inflammatory responses in circulating immune cells, and alters the systemic secretion profile of inflammatory factors and neurotransmitters. Upon infiltrating the cervical microenvironment, these circulating signals induce synchronous transcriptional abnormalities in homologous genes, thereby forming a neuroimmune axis that mediates the tumor-promoting effects of depression. A Venn intersection screening strategy was employed to eliminate background genes exhibiting tissue-specific expression variability, thereby retaining only those molecules demonstrating consistent dysregulation across both tissues. The intersecting genes identified through this approach concurrently regulate systemic stress responses and malignant tumor phenotypes, effectively minimizing transcriptional background noise from individual tissues. This method significantly enhances the precision of screening for key regulatory genes that serve as a link between the two diseases. Cluster analysis revealed that the DEGs related to the disease were significantly enriched in pathways associated with immune regulation, cell cycle, PD-1/PD-L1 signaling, and dopaminergic synapse. These findings closely align with the core hallmarks of cancer as summarized by Hanahan, which encompass immune evasion, uncontrolled proliferation, and metabolic reprogramming [16]. The dopaminergic synapse pathway primarily regulates the synthesis, metabolism, and intracellular signal transduction of monoamine neurotransmitters such as dopamine, serotonin, and norepinephrine. *MAOA* functions as a key rate-limiting enzyme in the degradation of these neurotransmitters, playing a dual biological role in maintaining neural homeostasis while facilitating tumor progression. Increasing evidence indicates that aberrant expression of *MAOA* serves as a critical molecular characteristic of MDD, with dysregulation directly disrupting systemic neurotransmitter homeostasis [17]. Furthermore, abnormal *MAOA* expression is extensively implicated in the progression of malignant tumors and acts as a key prognostic biomarker for various cancers, including triple-negative breast cancer [18]. *EZR* also exhibits dual regulatory roles in neural function and tumor progression. This protein influences depression-related behavioral phenotypes by altering the structure and function of astrocytes [19], while modulating the migration and chemosensitivity of CC cells, thereby contributing to malignant progression [20]. Prior research has demonstrated that cervical tumor epithelial cells consistently express two G-protein-coupled receptors, HTR2B and ADRB2, which specifically recognize and bind to peripherally accumulated serotonin and norepinephrine. Such interactions facilitate the transduction of peripheral neural signals into the tumor microenvironment [21, 22]. As a key membrane-cytoskeleton linker protein, *EZR* supports the stable membrane localization of these receptors, perpetuating the activation and amplification of the downstream FAK/ERK pro-metastatic signaling cascade, which enhances the malignant potential of tumors [23]. By integrating existing literature with our transcriptomic data, a novel neuro-oncogenic crosstalk pathway is proposed. In this model, depressive states trigger the downregulation of *MAOA* expression, significantly diminishing the degradation efficiency of peripheral monoamine neurotransmitters. This reduction leads to substantial accumulation of serotonin and norepinephrine in the circulatory system. These accumulated neurotransmitters subsequently interact with HTR2B and ADRB2 receptors located on the membranes of CC cells. The protein EZR plays a pivotal role in stabilizing the membrane distribution of these receptors, thus sustaining downstream oncogenic signaling pathways. This signaling cascade progressively amplifies malignant biological effects, ultimately facilitating the initiation and progression of CC.

Recent multi-omics research has established advanced frameworks for the exploration of tumor biomarkers and the construction of predictive models. A growing body of evidence suggests that multi-modal integrative modeling strategies, which incorporate multi-omics data, enhance feature stability [24]. Several investigations have developed gene signatures related to metabolism and immunity to elucidate the regulatory mechanisms underlying the remodeling of the tumor microenvironment [25]. Additionally, interpretable machine learning pipelines have been devised to identify robust and reliable molecular biomarkers for tumor prediction [26]. In contrast, some studies have identified candidate biomarkers based solely on differential expression analysis within single tumor cohorts [27]. Building on these methodological advancements, the current study employed LASSO regression in conjunction with multiple machine learning algorithms to identify core genes from intersecting DEGs between MDD and CC. As a result, four robust cross-disease biomarkers—*EZR*, *MAOA*, *CRAT*, and *CLIC*4—were successfully identified and used to construct a predictive signature. ROC analysis was conducted to preliminarily assess the diagnostic efficacy of a four-gene signature in identifying CC and MDD. In CC samples, the individual genes *CLIC4* and *CRAT* exhibited satisfactory discriminatory capabilities. The comprehensive four-gene signature achieved an internal AUC of 0.946, significantly surpassing the performance of individual genes and compensating for the limited specificity of single biomarkers, which aligns with previous research on multi-gene tumor signatures [28]. In the context of depressive disorder samples, individual genes demonstrated only modest discriminatory performance, with AUC values ranging from 0.607 to 0.668. However, the integration of the four-gene panel enhanced the AUC to 0.778, indicating that a multi-gene approach could improve diagnostic accuracy for depressive states, corroborating earlier findings on depressive biomarkers [29]. Notably, the robust internal diagnostic performance of *CLIC4* in CC is consistent with its biological role as an oncogene involved in remodeling the tumor microenvironment and mediating tumor immune suppression [30]. Notably, diagnostic results obtained from dataset GSE63514 were derived through internal validation within a limited cohort, comprising only 28 tumor specimens and 24 normal cervical tissues. The restricted sample size, along with the absence of an independent external validation set, heightens the risk of overfitting in machine learning modeling, which could potentially lead to an overestimation of the AUC values. Consequently, the current diagnostic performance of this four-gene signature is applicable solely to the internal dataset and cannot be generalized to broader populations. To facilitate clinical translation, validation using large independent clinical cohorts is essential for external verification and comprehensive evaluation.

This study conducted a systematic analysis of the core regulatory network associated with a four-gene signature (comprising *CLIC4*, *CRAT*, *EZR*, and *MAOA*) in the context of CC progression, utilizing GSEA. The results revealed that all four genes were significantly enriched in the apoptosis and DNA repair pathways, suggesting their potential role in promoting tumor cell survival by inhibiting apoptotic processes and maintaining genomic stability. Furthermore, certain genes were co-enriched in pathways such as the TGF-β signaling pathway and EMT. Previous studies have corroborated these findings, demonstrating a strong association between elevated *CLIC4* expression and both the TGF-β signaling pathway and EMT [30, 31]. The remodeling of the tumor microenvironment represents a fundamental mechanism underlying tumor initiation and progression, facilitated by a diverse array of functional coding genes that disrupt microenvironmental homeostasis. Prior multi-omics and cellular functional studies have confirmed that four categories of molecules—cytoskeleton regulators, ion channels, chromatin modulators, and phosphoesterases—play pivotal regulatory roles in tumor dynamics [32–35]. Building on this foundation, the current study conducts comprehensive bioinformatic analyses to explore the intrinsic relationships between these molecules and immune cell infiltration patterns in CC tissues, with the aim of identifying novel candidate targets for immunotherapy against CC. Utilizing the TIMER and CIBERSORT immune deconvolution algorithms for the analysis of bulk transcriptomic data, preliminary findings indicate a statistical correlation between *CLIC4* expression and tumor immune infiltration characteristics in CC. Elevated *CLIC4* expression in cervical lesions was positively associated with the abundance of M2-type tumor-associated macrophages (TAMs) and inversely correlated with the infiltration level of CD8^+^ cytotoxic T cells. Existing literature supports that M2-type TAMs secrete anti-inflammatory cytokines, such as IL-10 and TGF-β, which inhibit the activation of effector T cells and promote malignant tumor progression. Simultaneously, inadequate infiltration of CD8^+^ T cells compromises the body’s immune surveillance against tumor cells. Integrating these omics correlation findings, it is hypothesized that *CLIC4* may play a role in the M2 polarization of macrophages and the reduction of CD8^+^ T cell counts in lesions, potentially contributing to the establishment of an immunosuppressive tumor microenvironment that hinders control of CC. This potential immune-related correlation resembles the *CXCL8*-mediated immunosuppressive phenotype previously reported in colorectal cancer [36]. In contrast, elevated expression levels of the tumor suppressor gene *CRAT* were associated with increased infiltration of anti-tumor immune cells, including CD8⁺ T cells and NK cells, in CC tissues. This suggests that *CRAT* may exert tumor-suppressive effects by maintaining local immune activation. Previous reviews have summarized the prognostic significance of immune biomarkers such as PD-L1, tumor mutational burden (TMB), CD8^+^ tumor-infiltrating lymphocytes (TILs), and IL-6 in advanced CC, highlighting the limitations of relying on single biomarkers for clinical prediction. These findings suggest that multi-gene combined signatures may enhance the predictive accuracy of treatment responses [37]. *CRAT* and *CLIC4*, identified in our study, could serve as novel immune-associated markers, potentially augmenting the existing biomarker repertoire for CC.

Through an analysis of the GDSC drug sensitivity databases, this study has established, for the first time, that the expression levels of *MAOA*, *EZR*, *CRAT*, and *CLIC4* are significantly associated with the sensitivity of CC to various commonly utilized clinical drugs. These findings suggest that these genes may serve as potential biomarkers for predicting drug response and guiding personalized treatment strategies. Cobimetinib (GDC-0973), a highly selective MEK1/2 inhibitor already approved for melanoma and other cancer types [38], emerges as a potential candidate drug that targets pathways related to *CLIC4*, *EZR*, and *MAOA*. Additionally, *CLIC4* can facilitate tumor progression by modulating ERK/MAPK pathway activity [39]. This provides a theoretical foundation for the clinical screening of CC patients who may benefit from MEK inhibitor treatment, the selection of companion diagnostic markers, and the implementation of precise medication strategies.

Survival analysis indicated that elevated *CRAT* expression serves as a protective factor for patients with CC, while increased *CLIC4* expression correlates significantly with reduced OS, highlighting their considerable scientific research value and clinical application potential. In vitro experiments confirmed that *CRAT* is expressed at low levels in CC tissues. *CRAT* overexpression markedly inhibits the proliferation, colony formation, migration, and invasion of CC cells, thereby validating its tumor-suppressive role in CC. Consistent with previous findings, this result supports the notion that *CRAT* exerts a conserved tumor-suppressive effect in gynecological malignancies [40]. Mechanistically, *CRAT* remodels the malignant phenotypes of CC cells by regulating EMT. *CRAT* overexpression significantly upregulates the epithelial marker E-cadherin and downregulates the mesenchymal markers N-cadherin and Vimentin, as well as the key EMT transcription factor Snail1, thereby maintaining the epithelial characteristics of tumor cells and blocking EMT progression. These findings are consistent with earlier observations in ovarian cancer, further confirming that *CRAT* suppresses migration and invasion of gynecological tumor cells by reversing the EMT phenotype [40]. To further elucidate the upstream molecular mechanisms through which *CRAT* regulates EMT in CC, this study concentrated on the canonical IL-6/JAK/STAT3 signaling pathway. Previous research has established that aberrant activation of this pathway significantly induces EMT in CC, acting as a central oncogenic signaling axis that propels malignant progression [41]. Enrichment analysis confirmed that *CRAT* is significantly associated with the JAK/STAT3 pathway. Correlation analysis revealed a significant positive correlation between the expression level of *CRAT* and both *JAK1*, the upstream core kinase, and *STAT3*, the downstream effector molecule, within the JAK/STAT3 pathway. Considering the activation characteristics of this pathway, it can be inferred that CRAT may regulate the activity of the JAK1/STAT3 signaling axis. This regulation potentially contributes to the modulation of the EMT in tumor cells, thereby influencing the malignant progression of CC cells. As a member of the chloride channel family, *CLIC4* exhibits a cancer type-dependent role in tumorigenesis [42]. Previous studies have reported that *CLIC4* is downregulated in gastric and prostate cancers, inhibiting EMT progression to exert tumor-suppressive effects [43, 44]. In contrast, the present study confirms that *CLIC4* is markedly upregulated in CC tissues. Functional cellular assays demonstrated that silencing *CLIC4* significantly inhibits the proliferation, colony formation, migration, and invasion of HeLa and SiHa cells, indicating that *CLIC4* functions as a pivotal oncogene in CC. Mechanistic investigations revealed that *CLIC4* knockdown substantially elevates the expression of the epithelial marker E-cadherin while reducing levels of mesenchymal markers such as N-cadherin, Vimentin, and Snail1, thereby inducing a mesenchymal-epithelial transition (MET) in CC cells. Collectively, *CLIC4* promotes the invasion and metastasis of CC by activating the EMT signaling pathway, further supporting the role of EMT as a fundamental molecular mechanism driving the malignant progression and distant metastasis of CC [45]. Further mechanistic exploration centered on the TGF-β signaling pathway, a well-established upstream regulator of EMT. Accumulating evidence has illustrated that TGF-β-modulated *CLIC4* is essential for tumor microenvironment remodeling and metastatic competence in breast cancer [30]. In line with these observations, bioinformatic enrichment analysis revealed significant enrichment of *CLIC4* within the TGF-β signaling pathway, exhibiting strong positive correlations with core pathway components, specifically *TGFBR1*, and *SMAD2*. These findings lead to the hypothesis that *CLIC4* activates the TGF-β signaling cascade through the *TGFBR1*-*SMAD2* axis, which induces EMT and ultimately promotes malignant progression and metastasis of CC.

This study demonstrates that the four hub genes—*EZR*, *MAOA*, *CLIC4*, and *CRAT*—hold significant diagnostic potential and clinical translational value for CC patients experiencing depressive symptoms. However, the study is constrained by two primary limitations. Firstly, the elucidation of molecular mechanisms remains incomplete; only in vitro cellular experiments were conducted to explore the biological functions of *CLIC4* and *CRAT* in CC cells, without corroborative in vivo animal studies. Additionally, the synergistic regulatory mechanisms among the four core genes are not fully understood, which hinders a comprehensive interpretation of the molecular network linking depressive states to the progression of CC. Secondly, the study lacks sufficient clinical validation, as all gene screening and analyses were based solely on public transcriptomic datasets, without verification through large clinical cohorts, which diminishes the clinical applicability of the findings. Future research endeavors aim to enhance this study in two key areas by utilizing advanced multi-omics and multimodal integration methodologies. Firstly, multimodal frameworks such as CAMM and UniPET will be employed to integrate medical imaging, multi-omics sequencing data, and clinicopathological indicators [46, 47]. This approach seeks to develop a four-gene predictive signature with enhanced generalizability, facilitating the clinical translation of this molecular marker. Secondly, additional in vivo animal experiments will be conducted to systematically elucidate the interactive regulatory pathways of the four genes. Concurrently, clinical tissue samples will be collected for experimental validation. These enhancements are expected to establish a robust theoretical foundation for early screening, targeted therapy, and prognostic evaluation of CC patients exhibiting depressive symptoms.

## Conclusions

This study identified four core hub genes (*EZR*, *MAOA*, *CLIC4*, and *CRAT*) associated with cervical cancer. The four-gene signature constructed demonstrates high diagnostic efficacy and independent prognostic value for cervical cancer, while exhibiting moderate discriminatory potential for major depressive disorder. Bioinformatic analyses suggest that these genes are implicated in tumor metabolic reprogramming, cell cycle regulation, EMT, and remodeling of the tumor immune microenvironment. Furthermore, they correlate with the sensitivity to multiple anticancer drugs, with cobimetinib emerging as a potential targeted agent for pathways related to *EZR*, *MAOA*, and *CLIC4*. In vitro experiments further elucidated the contrasting roles of *CRAT* and *CLIC4*: *CRAT* inhibits CC malignancy and EMT via the JAK1/STAT3 pathway and is associated with a favorable prognosis, whereas *CLIC4* facilitates tumor progression and EMT through the TGF-β/TGFBR1/SMAD2 axis and is linked to adverse clinical outcomes. Collectively, this work provides a reliable gene signature for CC diagnosis and prognostic stratification, and elucidates the distinct mechanisms of two key regulatory genes, offering potential biomarkers and therapeutic targets for precise CC management.

## Supplementary Information

Supplementary Material 1.

Supplementary Material 2.

## Data Availability

All data supporting the findings of this study are available within the paper and its Supplementary Information.

## Abbreviations

CC: Cervical cancer
MDD: Major depressive disorder
GEO: Gene expression omnibus
DEGs: Differentially expressed genes
GO: Gene ontology
KEGG: Kyoto encyclopedia of genes and genomes
EV: Extracellular vesicle
EMT: Epithelial-mesenchymal transition
RMSE: Root mean square error
ROC: Receiver operating characteristic
AUC: Area under the curve
CI: Confidence interval
GSEA: Gene set enrichment analysis
MHC: Major histocompatibility complex
GDSC: Genomics of drug sensitivity in cancer
OS: Overall survival
RF: Random forest
DT: Decision tree
GBM: Gradient boosting machine
SVM: Support vector machine
DSS: Disease-specific survival
PFI: Progression-free interval
DFI: Disease-free interval
HPV: Human papillomavirus
TAMs: Tumor-associated macrophages
