# Supplementary material for: Screening of shared molecular markers between cervical cancer and major depressive disorder and the mechanism of CRAT/CLIC4 regulating EMT in cervical cancer cells
Source: BMC Cancer. 2026 Jul 20;26:972. doi: 10.1186/s12885-026-16571-5 (PMC13471574; doi:10.1186/s12885-026-16571-5)
Supplement: Supplementary file 1 [file 12885_2026_16571_MOESM2_ESM.pdf]

Figure 7B

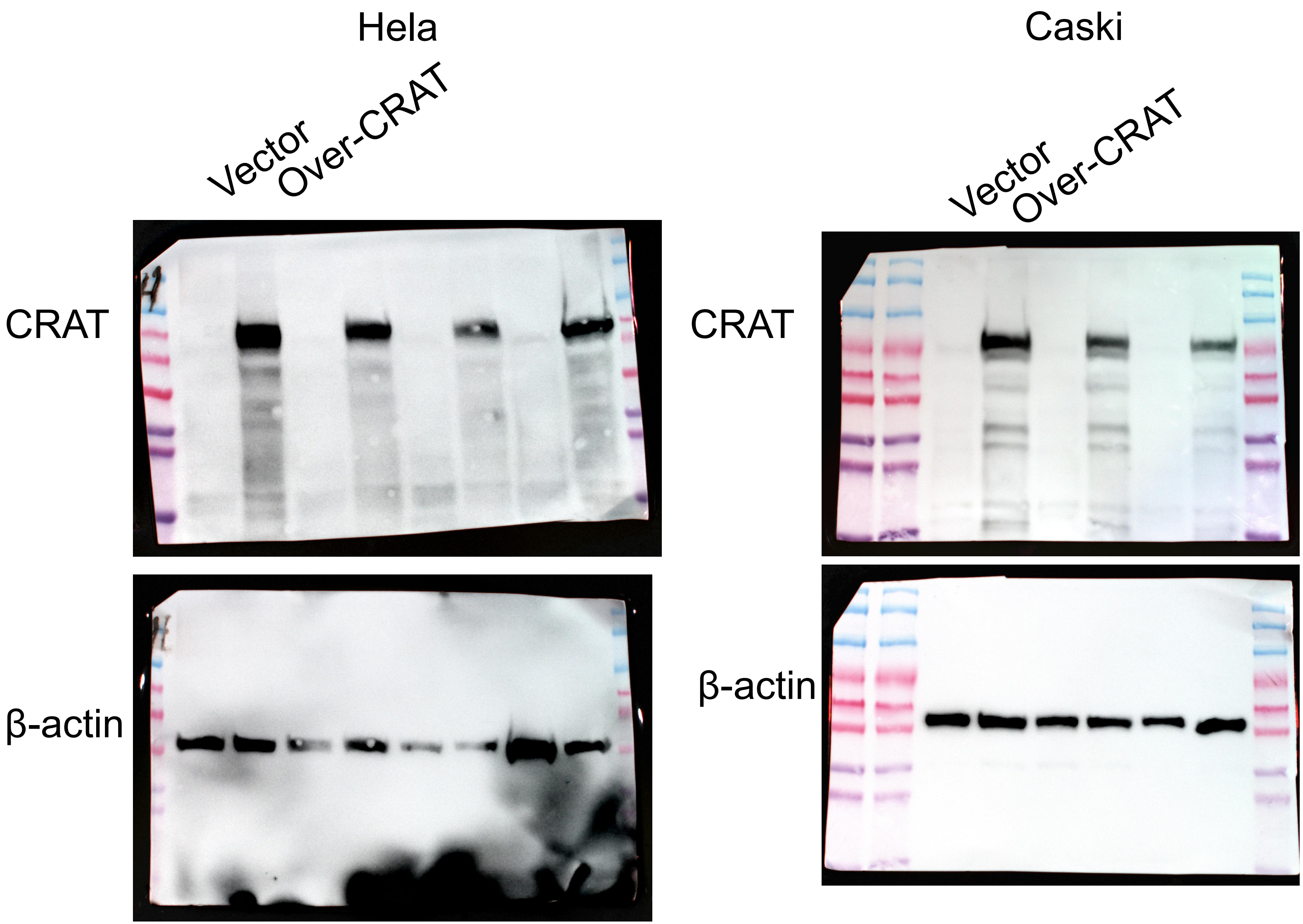

Hela
Vector
Over-CRAT
CRAT
β-actin
Caski
Vector
Over-CRAT
CRAT
β-actin

Figure 7G

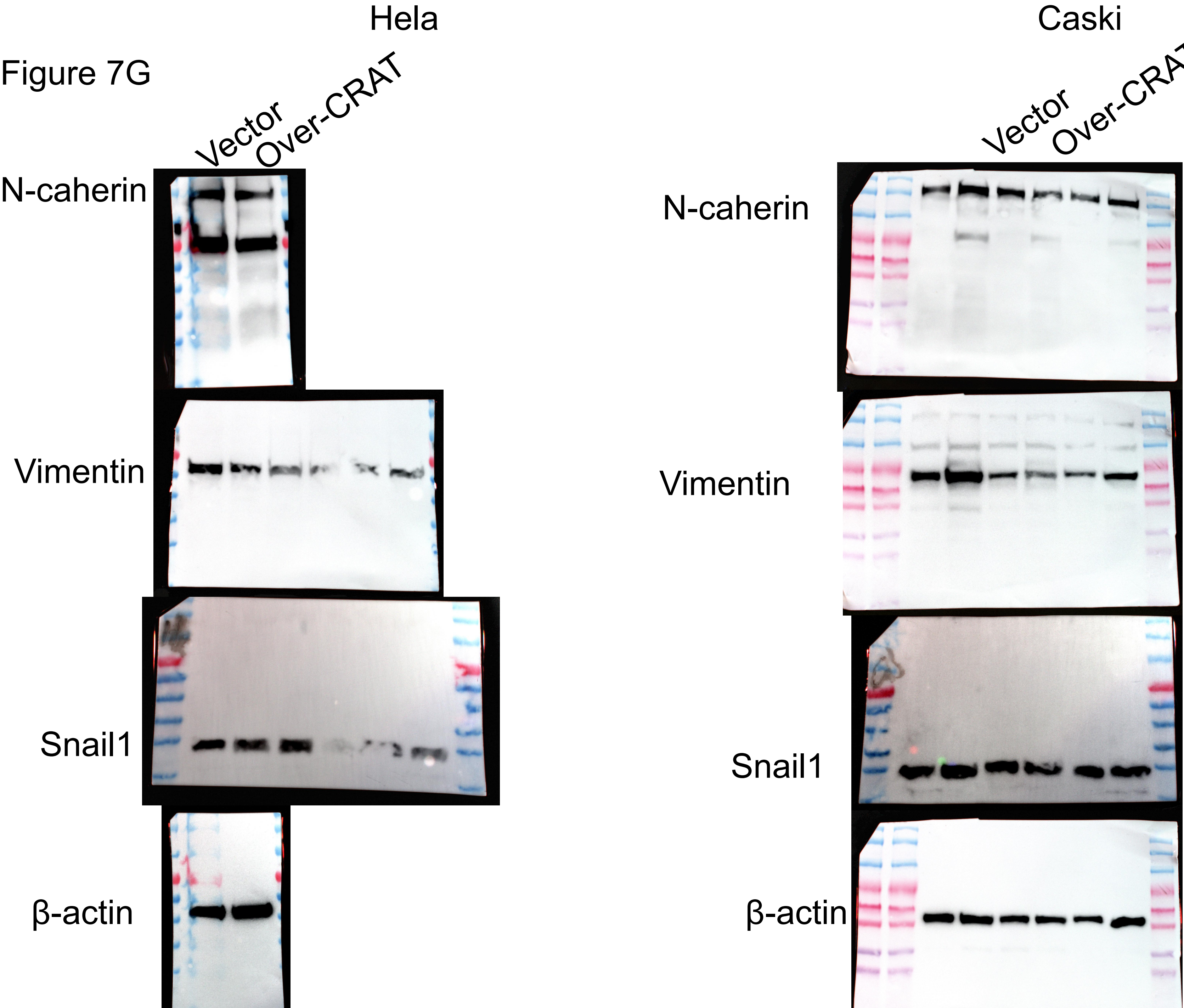
Hela
Caski
Vector
Over-CRAT
Vector
Over-CRAT
N-caherin
Vimentin
Snail1
β-actin
N-caherin
Vimentin
Snail1
β-actin

Figure 8A

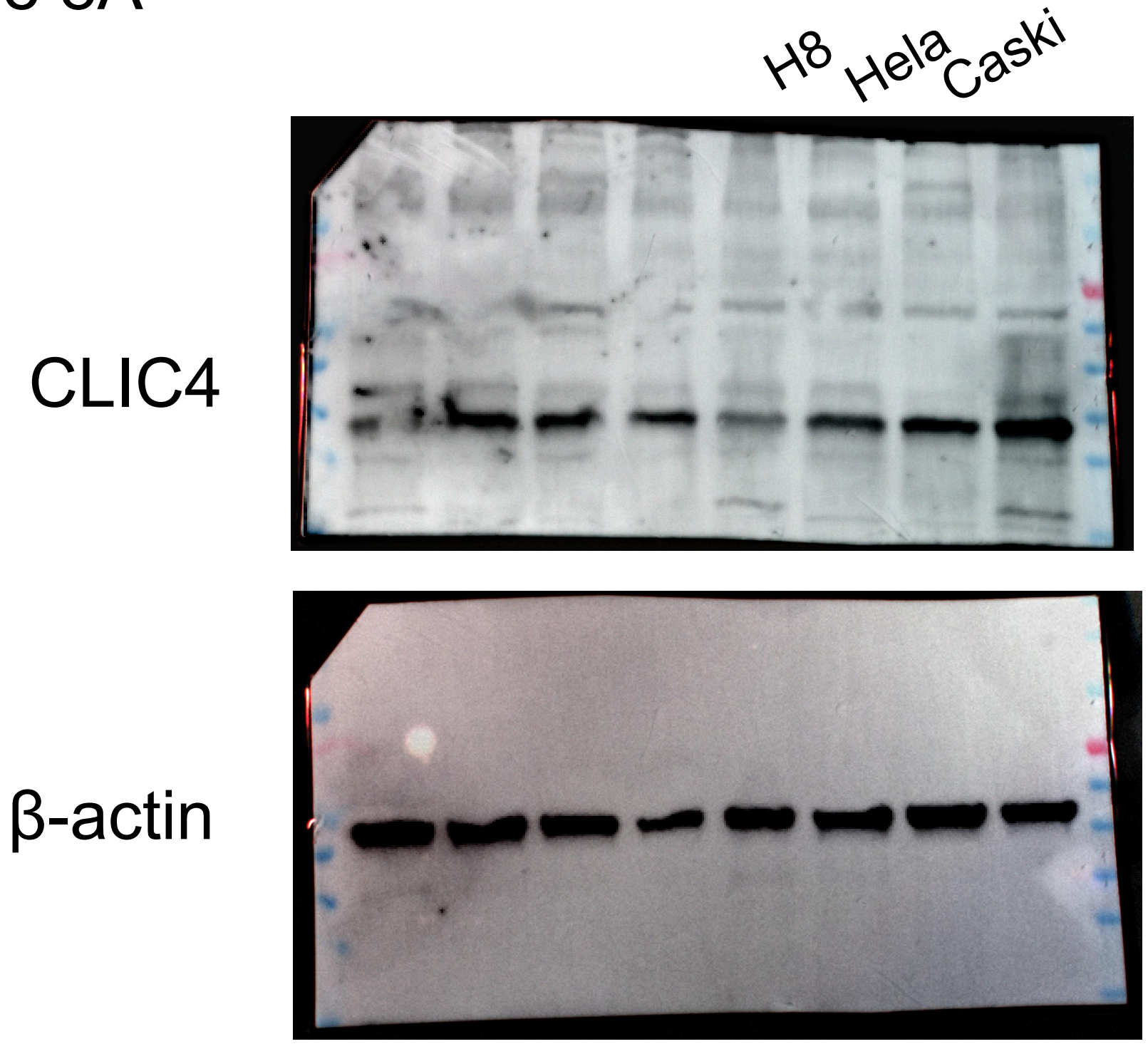

Figure 8C

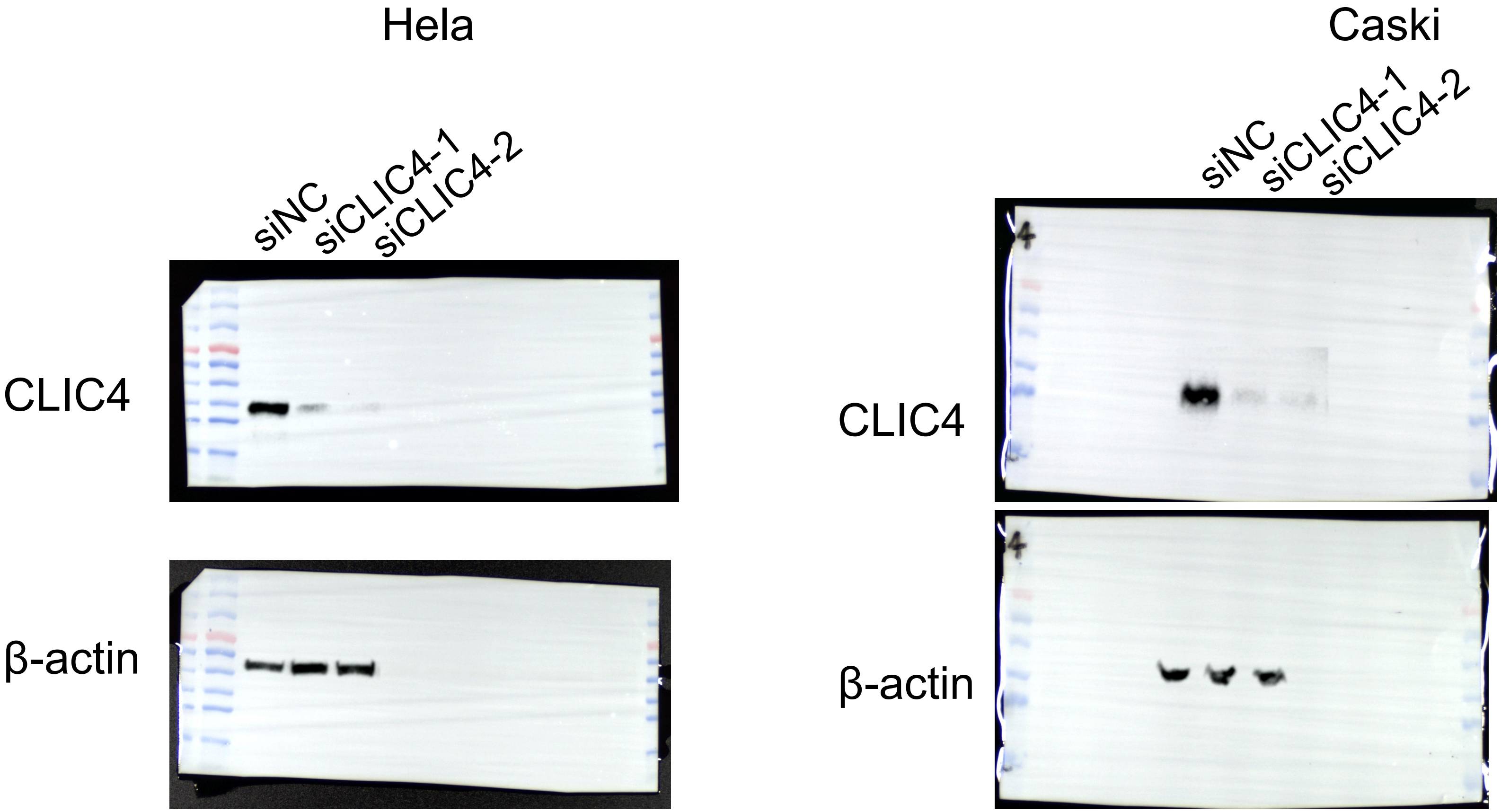
Hela
siNC
siCLIC4-1
siCLIC4-2
CLIC4
β-actin
Caski
siNC
siCLIC4-1
siCLIC4-2
CLIC4
β-actin

Figure 8G

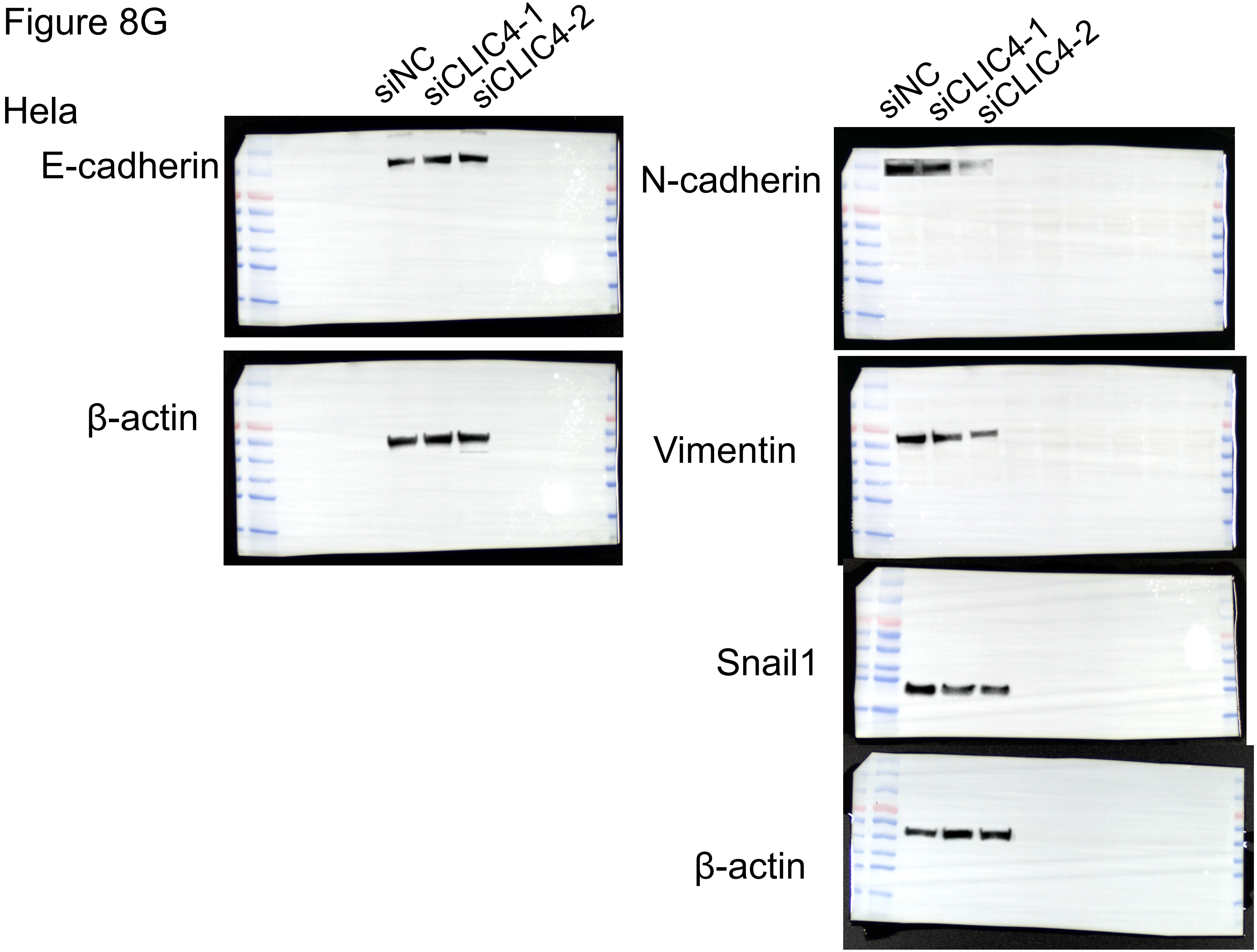

Hela
siNC
siCLIC4-1
siCLIC4-2
E-cadherin
β-actin
siNC
siCLIC4-1
siCLIC4-2
N-cadherin
Vimentin
Snail1
β-actin

Figure 8G

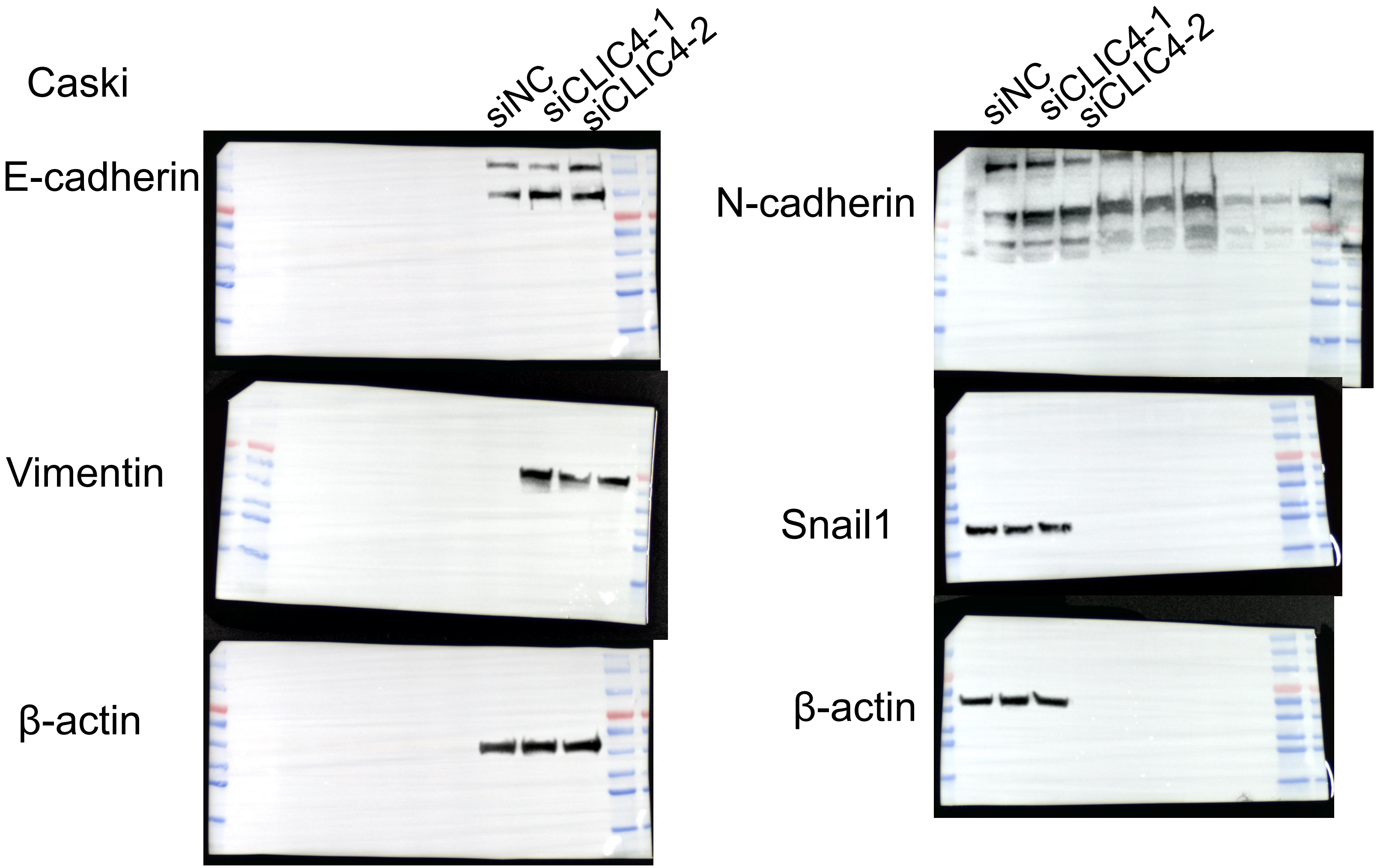
Caski
siNC
siCLIC4-1
siCLIC4-2
E-cadherin
Vimentin
β-actin
siNC
siCLIC4-1
siCLIC4-2
N-cadherin
Snail1
β-actin
