# Supplementary material for: Screening of shared molecular markers between cervical cancer and major depressive disorder and the mechanism of CRAT/CLIC4 regulating EMT in cervical cancer cells
Source: BMC Cancer. 2026 Jul 20;26:972. doi: 10.1186/s12885-026-16571-5 (PMC13471574; doi:10.1186/s12885-026-16571-5)
Supplement: Supplementary file 2 [file 12885_2026_16571_MOESM1_ESM.docx]

**Fig. S1** Correlation analysis of MAOA, EZR, CRAT, and CLIC4 expression with immune-related genes in CC. **(A)** Correlation heatmap between the four genes and chemokine genes in CC tumor tissue. **(B)** Correlation heatmap between the four genes and chemokine receptor genes in CC tumor tissue. **(C)** Correlation heatmap between the four genes and immune-activating genes in CC tumor tissue. **(D)** Correlation heatmap between the four genes and immunosuppressive genes in CC tumor tissue. **(E)** Correlation heatmap between the four genes and major histocompatibility complex (MHC) genes in CC tumor tissue.

**Fig. S2** The Venn diagram illustrates the element distribution and intersection characteristics of the molecular sets associated with the four genes CLIC4, CRAT, EZR, and MAOA.
